# Plasma cell targeting with the anti-CD38 antibody daratumumab in myalgic encephalomyelitis/chronic fatigue syndrome—a clinical pilot study

**DOI:** 10.3389/fmed.2025.1607353

**Published:** 2025-07-09

**Authors:** Øystein Fluge, Ingrid Gurvin Rekeland, Kari Sørland, Kine Alme, Kristin Risa, Ove Bruland, Karl Johan Tronstad, Olav Mella

**Affiliations:** ^1^The Cancer Clinic, Haukeland University Hospital, Bergen, Norway; ^2^Institute of Clinical Sciences, University of Bergen, Bergen, Norway; ^3^Department of Medical Genetics, Haukeland University Hospital, Bergen, Norway; ^4^Department of Biomedicine, University of Bergen, Bergen, Norway

**Keywords:** ME/CFS clinical trial, daratumumab, autoantibodies, plasma cell, feasibility, pathomechanism

## Abstract

**Background:**

Myalgic Encephalomyelitis/Chronic Fatigue Syndrome (ME/CFS) entails low quality of life for patients and massive societal costs. There is an urgent need for elucidation of disease mechanisms and for rational treatment. Our working hypothesis is that ME/CFS in a subgroup of patients is associated with functional autoantibodies emerging after an infection, and that plasma-cell depletion with transient reductions in serum immunoglobulins will have a beneficial effect on patients’ symptoms.

**Objective:**

To evaluate feasibility and toxicity of plasma-cell targeting treatment using the subcutaneous anti-CD38 antibody daratumumab (Darzalex^®^) in moderate to severe ME/CFS, and to assess the clinical course through 12–24 months follow-up after daratumumab intervention.

**Methods:**

We performed a prospective, open-label pilot trial (EudraCT 2022–000281-18). Ten female patients were enrolled. Following 12 weeks run-in, six patients received four daratumumab injections. The next four patients received four, followed by three additional injections from week 14.

**Results:**

All planned treatments were administered, and there were no serious adverse events. Four patients had no significant clinical changes. Six patients experienced marked improvement. For all 10 patients, mean SF-36 Physical Function (SF-36 PF) increased from 25.9 to 55.0 at 8–9 months (*p* = 0.002). DePaul Questionnaire-Short Form (DSQ-SF) symptom scores decreased from 72.3 to 43.1 (*p* = 0.002). In six responders, mean SF-36 PF increased from 32.2 to 78.3, and DSQ-SF score decreased from 71.1 to 24.3. Five of these six patients had major and sustained improvement with a mean SF-36 PF of 88 (range 80–95) toward end of follow-up. Mean steps per 24 h was 3,359 (range 1,493–6,277) at baseline. At 8–9 months, the mean number of steps was 5,862, and 7,392 in the six responders. All five patients with sustained improvement reached a mean step count above 10,000/24 h for some weeks, and above 15,000 on individual days. Relative reduction of serum IgG levels was 54% in six patients with clinical improvement, and 40% among four with no benefit. Low baseline NK-cell count in blood was significantly associated with lack of clinical response.

**Conclusion:**

Subcutaneous daratumumab in 10 ME/CFS patients was well tolerated. In six patients, treatment was associated with clinical improvement and concurrent transient reduction of serum IgG levels, indicating important pathomechanistic roles for long-lived plasma cells and functional autoantibodies. No definite conclusions should be drawn before a randomized study has been performed.

**Clinical trial registration:**

https://euclinicaltrials.eu, Identifier: 2022-000281-18.

## Introduction

Myalgic Encephalomyelitis/Chronic Fatigue Syndrome (ME/CFS) is a complex disease associated with high symptom burden and low quality of life (1–3). The societal costs are massive, and in the US the annual cost was estimated at 18–51 billion dollars (4).

The prevalence was estimated to 0.1–0.8% of the population (5, 6). ME/CFS is often preceded by an infection or other immunological trigger, is three to four times more prevalent in females than males, and is influenced by genetic predisposition (7).

The public awareness of ME/CFS has increased after the COVID-19 pandemic, with a proportion of patients struggling with brain fog, post-exertional malaise (PEM) and fatigue after the initial SARS-CoV-2 infection, denoted a post COVID-19 condition, or Long COVID (LC). Many patients recover, but among those with persistent LC for more than 2 years, approximately half meet the diagnostic criteria for ME/CFS (8, 9), indicating that this subgroup represents a post-infectious fatigue syndrome. Evidently, including this group will increase the prevalence, and in a recent large cross-sectional US survey, out of the 1.67% of adults who reported ME/CFS-like illness, 14% of cases were preceded by COVID-19 infection (10).

ME/CFS is presently a disease with unknown etiology, with no validated biomarker and no standard approved effective treatment. Patients experience less than consistent health services (11), and the lack of consensus on pathomechanisms may provide the ground for questionable and unscientifically founded advice and treatment of patients. There is an urgent need for elucidation of disease mechanisms and for development of effective treatment.

In 2021, we published a viewpoint article discussing pathomechanisms and possible intervention in ME/CFS (12). In brief, we suggested that ME/CFS could involve a variant of an autoimmune mechanism, with a role for B cells, plasma cells and autoantibodies. After systemic infections such as COVID-19 or Epstein–Barr virus (EBV), functional autoantibodies to a broad range of extracellular proteins emerge in otherwise healthy individuals as part of a normal immune response, including receptors and soluble molecules such as interferons, interleukins, and cytokines (13, 14).

Thus, it is plausible that the immune response after a systemic infection may induce a broad array of functional autoantibodies that affect biological systems and could be involved in the different symptoms experienced by ME/CFS patients. A role for G-protein coupled receptor (GPCR) autoantibodies has been suggested (15), including antibodies to muscarinic and adrenergic receptors, with subsequent dysregulation of autonomic function.

Such functional autoantibodies occur in previously healthy individuals after infections and usually subside over time, but in ME/CFS (or LC) some autoantibodies may persist. This model is compatible with a study of CFS following infectious mononucleosis, in which 13% of the infected fulfilled the criteria for CFS after 6 months, 7% after 12 months, and 4% after 2 years (16). However, although specific functional autoantibodies were elevated during acute COVID-19 infection (14), the number and magnitude of autoreactivities to the exoproteome were similar in individuals with or without ME/CFS (17), suggesting that the pattern of functional autoantibody responses, rather than single pathogenic autoantibodies, may be relevant.

Postinfectious functional autoantibody responses with disturbed regulation of blood flow may be a central pathomechanistic factor in ME/CFS, resulting in tissue hypoxia upon exertion. Endothelial dysfunction in small and large arteries has been shown (18–21). Studies have reported preload failure with reduced venous tone and venous return with low right atrial pressure during exercise tests, possibly due to autonomic dysregulation, followed by decreased cardiac output on exertion, and also reduced peripheral oxygen extraction with increased SvO_2_ in mixed venous blood (22, 23). Such physiological alterations with resultant tissue hypoxia on exertion, would be expected to induce autonomic compensation with increased sympathetic tone, which has been repeatedly observed in ME/CFS for decades (24, 25), as well as metabolic adaptations (26–28). Likewise, physiological functions like neuronal signaling may be affected.

Further, a role for autoantibodies in the ME/CFS pathomechanism is supported by data from immunoadsorption studies with clinical responses in ME/CFS patients (29, 30), and varying results in LC (31, 32). This technique extracts antibodies in general from the blood and transient symptomatic recovery can be seen within hours or days. However, the source of the antibody production is not affected.

From 2007 and onwards, we have observed patients in our cancer ward with long-standing ME/CFS who got cancer, and who reported that immunomodulatory cancer drug treatment they received had beneficial effects on the ME/CFS symptoms. We hypothesized that ME/CFS in a subgroup is associated with a variant of an autoimmune pathomechanism. Based on this hypothesis, we have performed several interventional clinical trials using the B-cell depleting anti-CD20 antibody rituximab in ME/CFS (33–37). Our early rituximab trials indicated that a subgroup of patients, and approximately half of those given rituximab maintenance treatment, showed signs of clinical improvement from B-cell depletion (36). However, the results from a larger randomized, double-blinded and placebo-controlled trial showed no difference between rituximab and placebo treatment (37). We also performed an open-label clinical trial with six infusions of the cytotoxic and immune system modulating drug cyclophosphamide in patients with moderate to severe ME/CFS, with clinical responses in 55%, and apparent benefit on the long-term clinical outcomes (33, 38). A recent review article has summarized ongoing trials and drugs of possible interest in ME/CFS and Post-COVID-19 condition (39).

Taking into account our observations from the clinical trials, we suggest that in ME/CFS patients the main autoantibody production occurs in long-lived plasma cells which, being CD20 negative, are not affected by the anti-CD20 antibody rituximab (40). This is a well-known mechanism for lack of response to rituximab in established autoimmune diseases, unless the treatment period is extended until the recruitment of new plasma cells is affected. We suspect this may have been the case in our previous open-label rituximab maintenance study (12, 36). Local observations of two ME/CFS patients with transient responses to the proteasome inhibitor bortezomib further supports the concept of plasma cell-directed treatment, however not pursued to a trial because of potential toxicity.

The known functions of long-lived plasma cells are well compatible with disease mechanisms we believe to be in ME/CFS. Once they have been programmed to produce autoantibodies, these plasma cells can survive in niches in the bone marrow or gut wall for decades and send a continuous stream of antibodies into the blood and further into different tissues.

Thus, we believe there is a rationale for therapeutic intervention targeting plasmablasts, short- and long-lived plasma cells in ME/CFS. An unselective plasma-cell depleting agent targeting CD38 will subsequently reduce both protective antibodies and autoantibodies, and could induce an altered balance of the plasma cell pool in some patients, potentially resulting in long-lasting changes in the disease phenotype upon repopulation and increasing IgG levels after intervention.

Herein we report the results of the KTS9 pilot study of 10 female patients with moderate to severe ME/CFS, treated with subcutaneous administration of the monoclonal anti-CD38 antibody daratumumab, targeting plasmablasts and long-lived plasma cells. The study is exploratory, and the main objectives were to assess safety and feasibility, and to explore possible beneficial or negative clinical effects on the course of ME/CFS through at least 12 months’, and up to 24 months of follow-up.

## Patients and methods

### Design overview

This prospective, open-label and single centre trial was performed at the Cancer Clinic at Haukeland University Hospital, Bergen, Norway. The objectives were to record feasibility and toxicity using subcutaneous daratumumab (Darzalex®) in patients with moderate to severe ME/CFS, and to assess the clinical course through follow-up for signs of clinical effects. Recruitment lasted from June 2022 to December 2023.

The protocol and amendment were approved by the Regional Committees for Medical and Health Research Ethics and by the Norwegian Medical Products Agency (EudraCT 2022–000281-18). The approved study protocol is available in Supplementary material. An independent Safety Board received reports on adverse events after the first two patients had completed intervention, and after the last treatment visits for patients 6 and 10. The trial was externally monitored by the section for monitoring at the Department for Research and Development at Haukeland University Hospital.

The inclusion criteria were ME/CFS according to Canadian consensus criteria (2003) (41) with moderate (mainly housebound) to severe (partly bedridden) disease, age 18–65 years, signed informed consent, duration of ME/CFS at least 2 years, and a defined onset, e.g., after an infection or other immunological trigger. Exclusion criteria are shown in the study protocol.

Serum immunoglobulins and lymphocyte subsets were measured in the hospital laboratory according to standard procedures, using nephelometry and flowcytometry with immunophenotyping, respectively.

### Intervention

All patients underwent a 3-months run-in period (weeks −12 to 0) before start of intervention, aiming to capture aspects of natural symptom variation over time without intervention. Daratumumab injections were administered in the outpatient ward at The Cancer Clinic, Haukeland University Hospital, at weeks 0, 2, 4 and 6 for all patients, with added maintenance treatments at weeks 14, 22 and 30 for the last four patients. Oral premedication was given at least 1 h before daratumumab injection. Before the first daratumumab injection: dexamethasone 10 mg orally, cetirizine 10 mg orally, paracetamol 1 g orally and montelukast 10 mg orally. Also, at least 20 min before daratumumab they received dexchlorpheniramine 5 mg iv. On the following 2 days patients took dexamethasone 4 mg orally. If patients experienced no adverse reaction to the first daratumumab injection, oral premedication before the subsequent injections was dexamethasone 10 mg, cetirizine 10 mg and paracetamol 1 g.

Daratumumab subcutaneous injections (Darzalex®) were given at a fixed dose of 1800 mg. The dose was injected over 3–5 min approximately 7.5 cm to the right or left of the umbilicus on intact, healthy skin.

The patient stayed in the outpatient ward for at least 6 h after the first Daratumumab injection. If the patient experienced no injection-related reactions (IRR) after the first injection, the observation period following the subsequent injections was 2 h.

### Self-reported questionnaires

Patients completed self-reported questionnaires every 2 weeks through the 3-months run-in period and until week 16 after start of treatment. Questionnaires were then administered every 4 weeks until week 40 for patients who had four treatments, and until week 60 for those who received maintenance injections, and at extended follow-up at 66 and 92 weeks. Questionnaires included the Norwegian-language versions of the Short Form-36 questionnaire for health-related quality of life (SF-36, ver. 1.2) (42), and the DePaul Symptom Questionnaire - Short Form (DSQ-SF) for ME/CFS symptoms (43). The Norwegian translation of DSQ-SF is based on the translation of the complete DePaul Symptom Questionnaire. Patients also recorded their perceived Function level in per cent according to a table with examples (Supplementary protocol).

### Fitbit activity armbands

Patients used a Fitbit Charge 5 activity armband through follow-up from week −12 to 40, continuously recording steps per 24 h and resting heart rate. A Data Protection Impact Assessment was performed prior to study start. To protect the participants’ privacy, we used pseudonymization toward third parties. Each participant Fitbit account was set up using a study-specific e-mail address, initials instead of name and a fictitious date of birth. Fitbit’s terms of use complied with the General Data Protection Regulation (GDPR) directive. Fitbit activity data from each participant were downloaded at the study centre regularly, using the Fitbit web API.^1^ For each participant we registered an Oauth 2.0 application with type set as “personal.” The scopes were set to heartrate + sleep + activity.

An R-script was generated to facilitate downloading of data from all participants. For more detailed information on data protection issues see trial protocol (Supplementary protocol).

### Statistics

As the first known experience with plasma-cell depletion in ME/CFS, this pilot study was deliberately descriptive, and no criteria for response or non-response were predefined in the protocol. Adverse events of grade two or higher were summarized according to CTCAE ver. 5.0. The outcome measures were based on DSQ-SF score, SF-36 Physical Function (SF-36 PF), perceived Function level, and measured Steps per 24 h.

The courses of the clinical variables SF-36 PF, DSQ-SF total score and Function level were described by the raw data for the individual patients, and for the pooled group of patients by means with 95% CI. The characterization of patients with clinical improvement or no improvement during follow-up, was based on the clinical assessment at investigator visits in addition to patient-reported measures and Fitbit data.

General Linear Model (GLM) repeated measures was used to assess the changes in outcome variables (SF-36 PF, DSQ-SF total score, Function level) over time, from baseline through follow-up, with *p*-values for the time effect. The p-values for each time level were assessed by simple contrasts in the time domain, as compared to baseline. Greenhouse–Geisser corrections were used for the General Linear Model analyses with multiple levels of the dependent variable due to violations of the sphericity assumption, i.e., the variances of the differences between every pair of time points were not equal. The baseline values comprised means of seven recordings from week −12 to week 0 (run-in period, before start of intervention).

Correlation analyses (Spearman’s rho) were performed between baseline number of NK cells and increase in SF-36 PF from baseline to weeks 36–40, and between baseline number of NK cells and maximum percent reduction of serum IgG during follow-up. The analyses were performed using IBM SPSS Statistics ver.29 (IBM Corp., Armonk, USA), and Graphpad Prism ver.10 (GraphPad Software, La Jolla, USA).

## Results

### Study population

**Figure 1:**
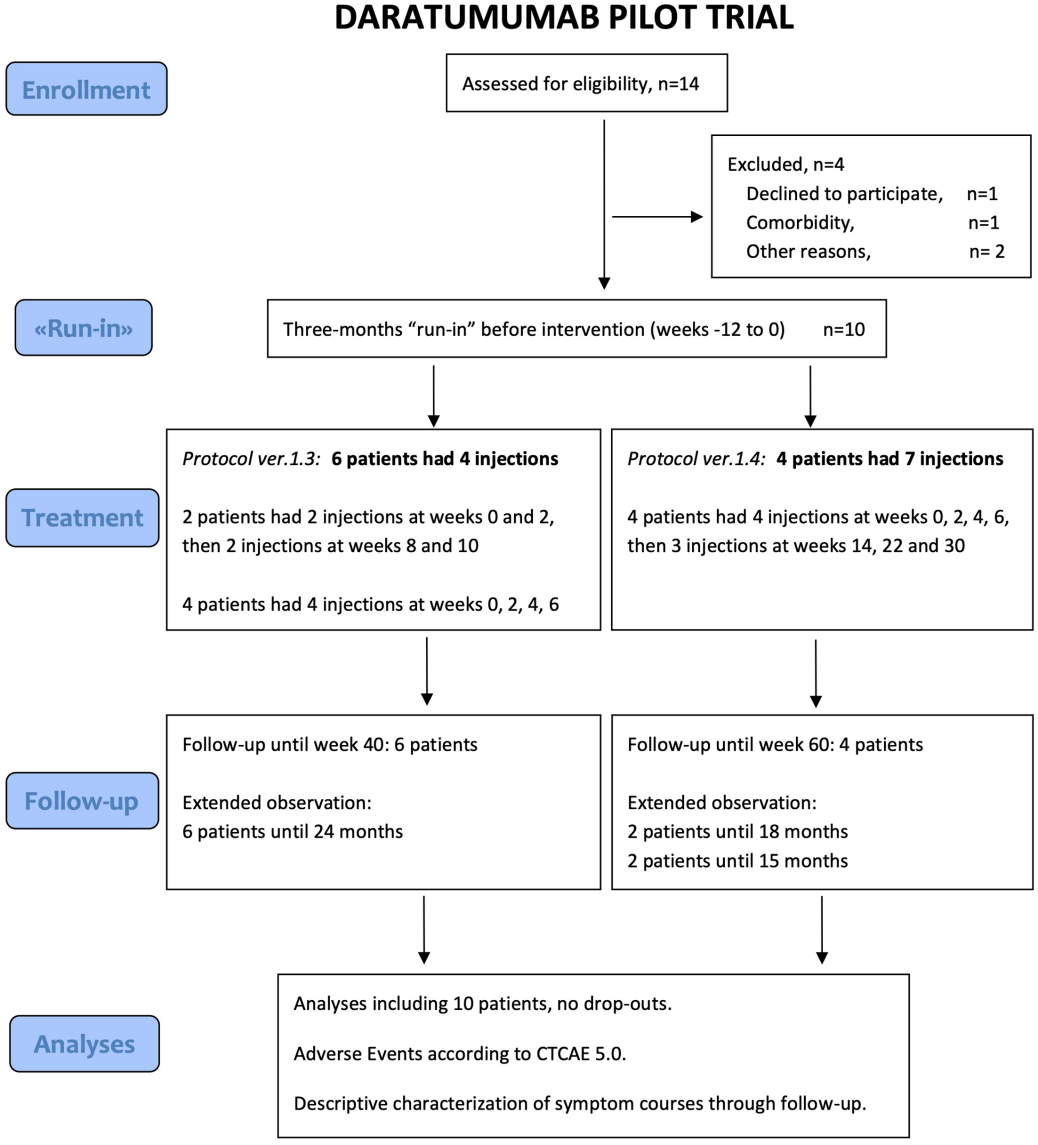
Consort Flow Diagram of inclusion, intervention and follow-up.

The flow chart for screening, inclusion, run-in, intervention and follow-up is shown in Figure 1.

Fourteen patients were screened for eligibility. Six patients were included in the time period June 2022 to January 2023. After the 3-months run-in, the first two patients each received two Darzalex injections 2 weeks apart, followed by 6 weeks observation to exclude any initial unexpected side effects, and then two additional injections 2 weeks apart. The next four patients received four injections 2 weeks apart followed by observation.

After observation of these first six patients, no serious adverse events (SAE) were recorded. Two patients experienced sustained and marked clinical improvement, and one a more transient, moderate improvement. To further explore dose–response relationships, an amendment was filed and four additional patients were included from June 2023 to December 2023. These four received the initial four injections 2 weeks apart and, provided there were any clinical signs of benefit 6 weeks after the fourth injection, a further three additional injections at weeks 14, 22 and 30.

All 10 patients received the planned number of subcutaneous Darzalex injections, in total 52. The first six patients completed follow-up from week −12 to week 40, and added follow-up visits according to protocol amendment at weeks 66 and 92. The last four patients completed follow-up from week −12 to week 48, and have yet to complete extended follow-up.

Table 1 shows baseline characteristics for all included 10 female patients, with a mean age of 38 years (range 20–61 years), and disease duration of mean 12 years (median 11 years, range 3–35 years). All 10 patients had a defined ME/CFS disease onset, in nine out of 10 preceded by an immunological trigger, usually infection. Baseline characteristics are also shown separately for the group of six patients with clinical improvement during follow-up, and the group of four patients with no improvement (Table 1). One patient had previously participated in the cyclophosphamide trial with inclusion in 2016 (33), while the other nine had not participated in any clinical study aimed at treating ME/CFS. None of the patients received any alternative intervention aimed at treating ME/CFS during the trial. They were treated separately, asked not to seek contact with other trial patients and not to communicate their symptom development on social media. The 10 included patients had no relevant comorbidity. Except for one 61-year-old woman, the other nine were premenopausal at inclusion. None of the included patients had Long COVID.

**Table 1:** Baseline characteristics of the KTS9 daratumumab pilot study population.

Characteristic	All patients *n* = 10	Clinical improvement^a^ *n* = 6	No improvement^b^ *n* = 4
Female, *n* (%)	10 (100)	6	4
Age (years), mean (min-max)	38 (20–61)	38 (20–61)	38 (22–50)
BMI^c^, mean (min-max)	25.9 (19.7–32.9)	27.6 (21.7–32.9)	23.2 (19.7–28.4)
ME/CFS disease duration (years), mean (min-max)	12 (3–35)	15 (3–35)	9 (5–11)
ME/CFS disease severity^d^			
Moderate, *n* (%)	7 (70)	5	2
Moderate/severe, *n* (%)	1 (10)	1	0
Severe, *n* (%)	2 (20)	0	2
Immune trigger upfront ME/CFS^e^, *n* (%)	9 (90)	6 (100)	3 (75)
DSQ-SF total score^f^ 3 months run-in, mean (min-max)	72.3 (60.3–87.1)	71.1 (60.3–82.6)	74.0 (66.2–76.9)
SF-36 physical function^g^, 3 months run-in, mean (min-max)	25.9 (5.0–45.7)	32.0 (10.0–45.7)	16.6 (5.0–35.0)
Function level (%)^h^, 3 months run-in, mean (min-max)	15.6 (9.3–21.7)	17.7 (11.7–21.7)	12.3 (9.3–15.5)
Steps, mean per 24 h^i^, 3 months run-in, mean (min-max)	3,359 (1493–6,277)	3,363 (1760–5,497)	3,353 (1493–6,277)
Serum IgG (g/L)^j^, mean (min-max)	9.9 (6.8–13.2)	10.2 (7.5–13.2)	9.6 (6.8–10.2)
Serum IgA (g/L)^k^, mean (min-max)	2.3 (0.8–3.4)	2.7 (2.2–3.4)	1.9 (0.8–2.7)
Serum IgM (g/L)^l^, mean (min-max)	1.3 (0.3–2.0)	1.1 (0.3–1.6)	1.6 (1.2–2.0)
CD19-pos. B-cells (x10^6^/L)^m^, mean (min-max)	227 (43–459)	251 (43–459)	191 (82–320)
NK-cells (x10^6^/L)^n^, mean (min-max)	181 (80–383)	238 (136–383)	97 (80–115)

^a^Patients with clinically assessed significant improvement during follow-up. ^b^Patients with no clinically assessed significant improvement. ^c^Body Mass Index (kg/m^2^). ^d^Clinically assessed ME/CFS severity. ^e^Self-reported infection or vaccine prior to onset of ME/CFS disease. ^f^De Paul Questionnaire – Short Form (DSQ-SF) total score, scale 0–112, lower number denotes less symptoms, mean of seven recordings during 3 months’ run-in. ^g^Short Form-36 Physical Function, scale 0–100, higher number denotes better physical function, mean of seven recordings during 3 months’ run-in. ^h^Baseline self-reported Function level, scale 0–100%, mean of seven recordings during 3 months’ run-in. ^i^Steps per 24 h, mean of days 1–84. ^j^Baseline serum IgG, normal range 6.0–12.0 g/L. ^k^Baseline serum IgA, normal range 0.8–4.9 g/L. ^l^Baseline serum IgM, normal range 0.3–2.3 g/L. ^m^Baseline number of CD19 positive B cells (normal range 88–581 ×10^6^/L). ^n^Baseline number of CD56/16 positive NK cells (normal range 59–533 ×10^6^/L).

### Clinical outcome measures

**Figure 2:**
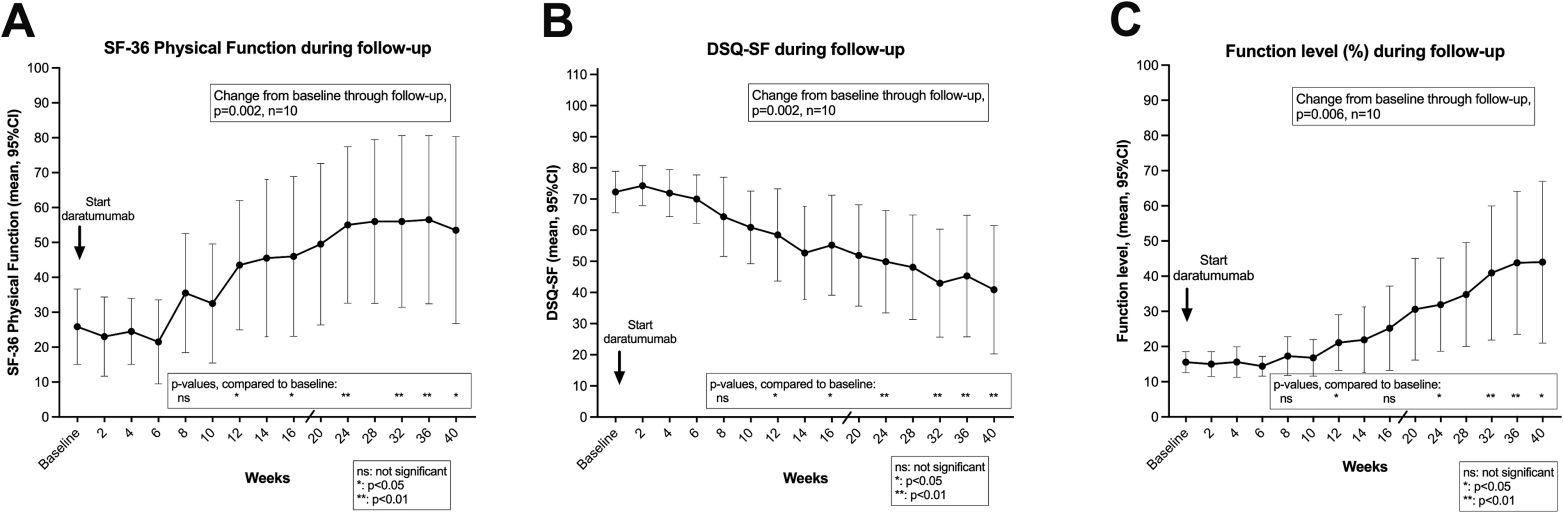
Short form-36 physical function (SF-36 PF) **(A)**, De Paul Questionnaire – short form (DSQ-SF) total score **(B)**, and function level **(C)** through follow-up. In **(A–C)** means with 95% CI at different time points through 40 weeks follow-up, after start of daratumumab intervention. The baseline values are the means of seven recordings from week −12 to week 0 (run-in period), before start of intervention. *p*-values from General Linear Model repeated measures assessing changes in the outcome variable from baseline through follow-up. The *p*-values for each time point were assessed by the contrasts for the time effect, as compared to baseline. *p*-values, ns: not significant; *: <0.05; **: <0.01. SF-36 PF, scale 0–100, higher value denotes better self-reported physical function. DSQ-SF total score, scale 0–112, higher value denotes worse ME/CFS symptoms. Function level, scale 0–100, higher value denotes better function.

The clinical courses for the 10 patients included in the daratumumab pilot trial are shown in Figures 2A–C, showing the means (and 95% CI) for SF-36 Physical Function, DSQ-SF total score and Function level, through 40 weeks from start of intervention. As detailed in Table 2, the mean values for SF-36 PF increased from a score of 25.9 at baseline to mean 55.0 in the time interval 8–9 months after start of intervention. Correspondingly, the average of DSQ-SF symptom scores decreased from 72.3 at baseline to 43.1, and Function level increased from 15.6 to 42.8%. The differences in outcome measures over time as compared to baseline were significant, assessed by General Linear Model repeated measures (*p* = 0.002, 0.002 and 0.006, for SF-36 PF, DSQ-SF and Function level, respectively).

**Table 2:** Clinical and laboratory data during follow-up.

Characteristic	All patients *n* = 10	Clinical improvement^a^ *n* = 6	No improvement^b^ *n* = 4
DSQ-SF total score^c^ Baseline–mean 8/9 months Difference, mean (min-max)	72.3–43.1 29.2 (−2.7–68.6)	71.1–24.3 46.8 (27.6–68.6)	74.0–71.3 2.7 (−2.7–5.8)
SF-36 Physical Function^d^, Baseline–mean 8/9 months Difference, mean (min-max)	25.9–55.0 29.1 (0.0–58.6)	32.0–78.3 46.3 (11.1–58.6)	16.6–20.0 3.4 (0.0–10.7)
Function level (%)^e^, Baseline–mean 8/9 months Difference, mean (min-max)	15.6–42.8 27.2 (−1.4–67.9)	17.7–62.4 44.7 (4.3–67.8)	12.3–13.4 1.1 (−1.4–6.8)
Steps, mean per 24 hours^f^, Baseline–mean 8/9 months Difference, mean (min-max)	3,359–5,862 2,503 (−875–6,589)	3,663–7,393 3,730 (−385–6,589)	3,353–3,566 213 (−875–1,386)
Serum IgG (g/L)^g^, Baseline–2 to 5–9 months	9.9–5.9 to 5.5–5.6	10.2–5.7 to 5.1–5.1	9.6–6.3 to 6.2–6.3
Serum IgA (g/L)^h^, Baseline–2 to 5–9 months	2.3–0.6 to 0.7–0.9	2.7–0.7 to 0.7–0.9	1.7–0.6 to 0.7–0.9
Serum IgM (g/L)^i^, Baseline–2 to 5–9 months	1.3–0.6 to 0.7–0.9	1.1–0.5 to 0.6–0.7	1.6–0.9 to 1.0–1.2
CD19-pos. B cells (x10^6^/L)^j^, Baseline–2 to 5–9 months	227–245 to 227 - 246	250–303 to 271–267	191–157 to 172–204
NK cells (x10^6^/L)^k^, Baseline–2 to 5–9 months	182–47 to 44–53	238–60 to 55–54	97–27 to 30–51
Serum IgG1 (g/L)^l^, Baseline–9 months	6.38–3.58	6.50–3.23	6.20–4.11
Serum IgG2 (g/L)^m^, Baseline–9 months	3.11–1.91	3.13–1.62	3.09–2.10
Serum IgG3 (g/L)^n^, Baseline–9 months	0.41–0.18	0.40–0.16	0.43–0.20
Serum IgG4 (g/L)^o^, Baseline–9 months	0.42–0.17	0.60–0.21	0.14–0.10

^a^Patients with clinically assessed significant improvement during follow-up. ^b^Patients with no clinically assessed significant improvement. ^c^De Paul Questionnaire – Short Form (DSQ-SF) total score, scale 0–112, lower number denotes less symptoms, mean of seven recordings during 3 months’ run-in, and mean of recordings at 8 and 9 months after start of intervention. ^d^Short Form-36 Physical Function subscale, scale 0–100, higher number denotes better physical function, mean of seven recordings during 3 months’ run-in, and mean of recordings at 8 and 9 months after start of intervention. ^e^Self-reported Function level, scale 0–100%, mean of seven recordings during 3 months’ run-in, and mean of recordings at 8 and 9 months. ^f^Steps per 24 hours, mean of run-in days 1–84, and mean of recordings days 309–365 after inclusion. ^g^Serum IgG levels, mean at baseline, 2, 5 and 9 months after start of intervention, normal range 6.0–12.0 g/L. ^h^Serum IgA levels, mean at baseline, 2, 5 and 9 months after start of intervention, normal range 0.8–4.0 g/L. ^i^Serum IgM levels, mean at baseline, 2, 5 and 9 months after start of intervention, normal range 0.3–2.3 g/L. ^j^CD19 positive B cells, mean at baseline, 2, 5 and 9 months after start of intervention, normal range 88–581 × 10^6^/L. ^k^CD56/16 positive NK cells, mean at baseline, 2, 5 and 9 months after start of intervention, normal range 59–533 × 10^6^/L. ^l^Serum IgG1, mean at baseline and 9 months, normal range 5.2–12.7 g/L. ^m^Serum IgG2, mean at baseline and 9 months, normal range 1.43–5.60 g/L. ^n^Serum IgG3, mean at baseline and 9 months, normal range 0.28–1.05 g/L. ^o^Serum IgG4, mean at baseline and 9 months, normal range 0.08–1.04 g/L.

**Figure 3:**
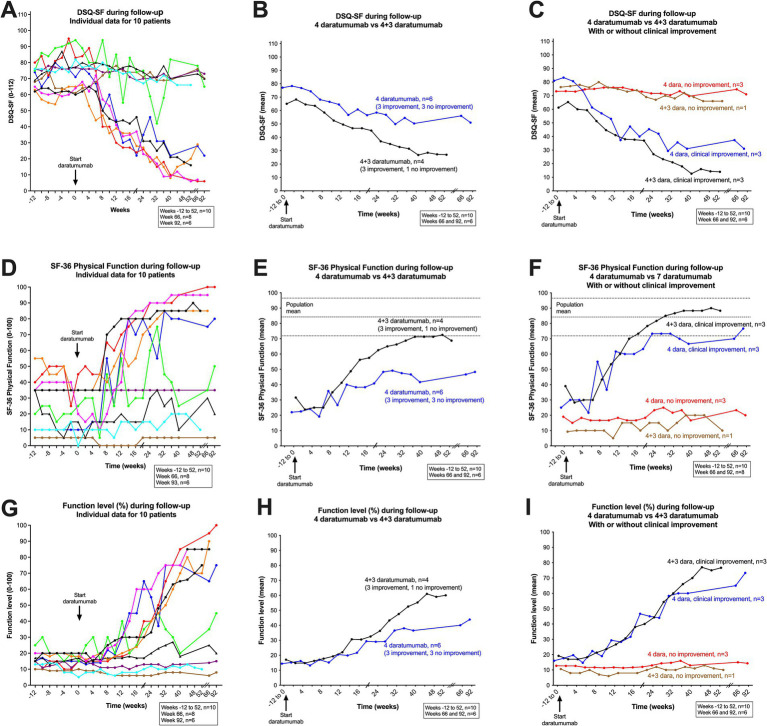
DSQ-SF, total score **(A–C)**, SF-36 Physical Function **(D–F)** and Function level **(G–I)**, individual data for 10 patients through follow-up. In **(A)** (DSQ-SF total score), **(D)** (SF-36 PF), and **(G)** (Function level), the raw data are shown, from week −12 through the run-in period before intervention to week 0, and then to week 92 after start of intervention (patients 1–6), and to week 52 (patients 7–10). In **(A,D,G)** each patient is represented by a line with a defined color, which is consistent through all figures and supplemental figures in the article. **(B)** (DSQ-SF total score), **(E)** (SF-36 PF) and **(H)** (Function level), show the means at each time point, for the group of six patients who received four daratumumab injections (blue color), and the group of four patients who received 4 + 3 daratumumab injections (black color), are shown. In **(C)** (DSQ-SF total score), **(F)** (SF-36 PF), and **(I)** (Function level), the groups of six patients who received four daratumumab injection, is further divided into patients with clinical improvement (*n* = 3, blue) or with no improvement (*n* = 3, red). Correspondingly, the group of four patients who received 4 + 3 daratumumab injection is divided into those with a clinical improvement (*n* = 3, black), or with no improvement (*n* = 1, brown). SF-36 PF, scale 0–100, higher value denotes better physical function. DSQ-SF total score, scale 0–112, higher value denotes worse ME/CFS symptoms. Function level, scale 0–100, higher value denotes better function.

**Figure 4:**
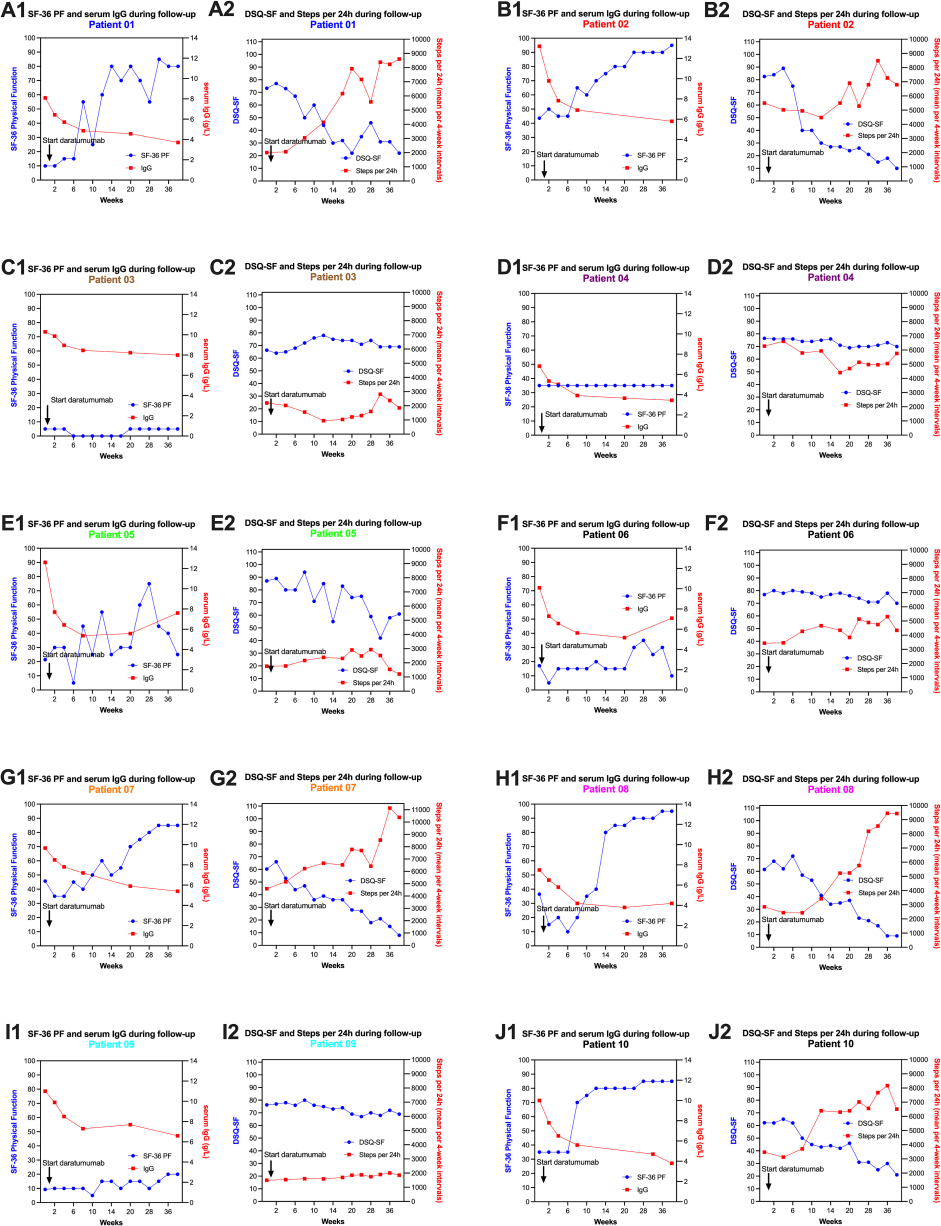
SF-36 PF, DSQ-SF, steps per 24 h and serum IgG levels during follow-up, for each of the 10 patients included in the daratumumab pilot study. Each patient has two panels (e.g., **A1,A2** for patient no. 1, **B1,B2** for patient no. 2, **C1,C2** for patient no. 3, **D1,D2** for patient no. 4, **E1,E2** for patient no. 5, **F1,F2** for patient no. 6, **G1,G2** for patient no. 7, **H1,H2** for patient no. 8, **I1,I2** for patient no. 9, **J1,J2** for patient no. 10). Panels no. 1 for each patient shows the data for SF-36 PF and serum IgG levels; panel no. 2 shows DSQ-SF and Steps per 24 h (means per 4-week intervals). The baseline data for SF-36 PF and DSQ-SF are the means of seven recordings through the run-in period before intervention, from week −12 to 0. The baseline data for steps per 24 h are the means for days 1-84.

Interestingly, for the group of six patients with a clinically assessed improvement during follow-up, the mean SF-36 PF score increased from baseline 32.2 to 78.3, and the DSQ-SF score decreased correspondingly from 71.1 to 24.3 (Table 2). Out of this group of six patients with improvement after daratumumab intervention, five patients had a sustained clinical improvement until end of follow-up (i.e., 52–104 weeks from inclusion). For these five patients, the SF-36 PF values in the last part of follow-up were close to or within the general population range (80, 95, 85, 95, 85, respectively) (Figure 3D). One patient (represented by a green line in figures) had a moderate and transient improvement with an increase of SF-36 PF from baseline 21.4 to maximum 75. However, this was followed by a partial relapse toward the end of follow-up, with mean SF-36 PF 32.5 at 8–9 months (Figures 3D, 4E1,E2). The increases in function, recorded as SF-36 PF and Function level, and decreases in symptoms, recorded as DSQ-SF, as calculated by difference from baseline to mean at 36–40 weeks, are shown in Table 2 and in Supplementary Figure 1.

The individual data for these self-reported clinical variables are shown in Figures 3A,D,G (the color codes for the lines representing each patient are identical in all figures). Figures 3B,E,H show the mean values for the three clinical variables SF-36 PF, DSQ-SF and Function level, grouped by the six patients who received four daratumumab injections versus four patients who received four + three daratumumab injections. Figures 3C,F,I show the mean values by number of administered daratumumab injections as above, but further divided into patients with clinical improvement versus no improvement. Interestingly, the patterns for clinical improvement were relatively homogenous, as all six patients who experienced clinical benefit started to improve during the time interval 6–8 weeks after the first daratumumab injection (Figures 3A,D,G).

Conversely, the group of four patients with no clinical improvement had very stable values for SF-36 PF, DSQ-SF and Function level through follow-up, e.g., with means for SF-36 PF 16.6 at baseline and 20.0 at 8–9 months (Figures 3, 4 and Table 2).

The details for each patient, from start of intervention through follow-up to week 40, for SF-36 PF, serum IgG levels, DSQ-SF total score and Steps per 24 h, are shown in Figures 4A1–J2.

Generally, the group of six patients who experienced clinical benefit during follow-up reported that the intervention affected all the characteristic ME/CFS symptoms. This is also evident from the different SF-36 subscales during follow-up (Bodily Pain, Vitality, Social Function, Physical Function, General Health), shown in Supplementary Figure 2, by individual and by pooled data (means and 95% CI).

### Steps per 24 h and resting heart rate

**Figure 5:**
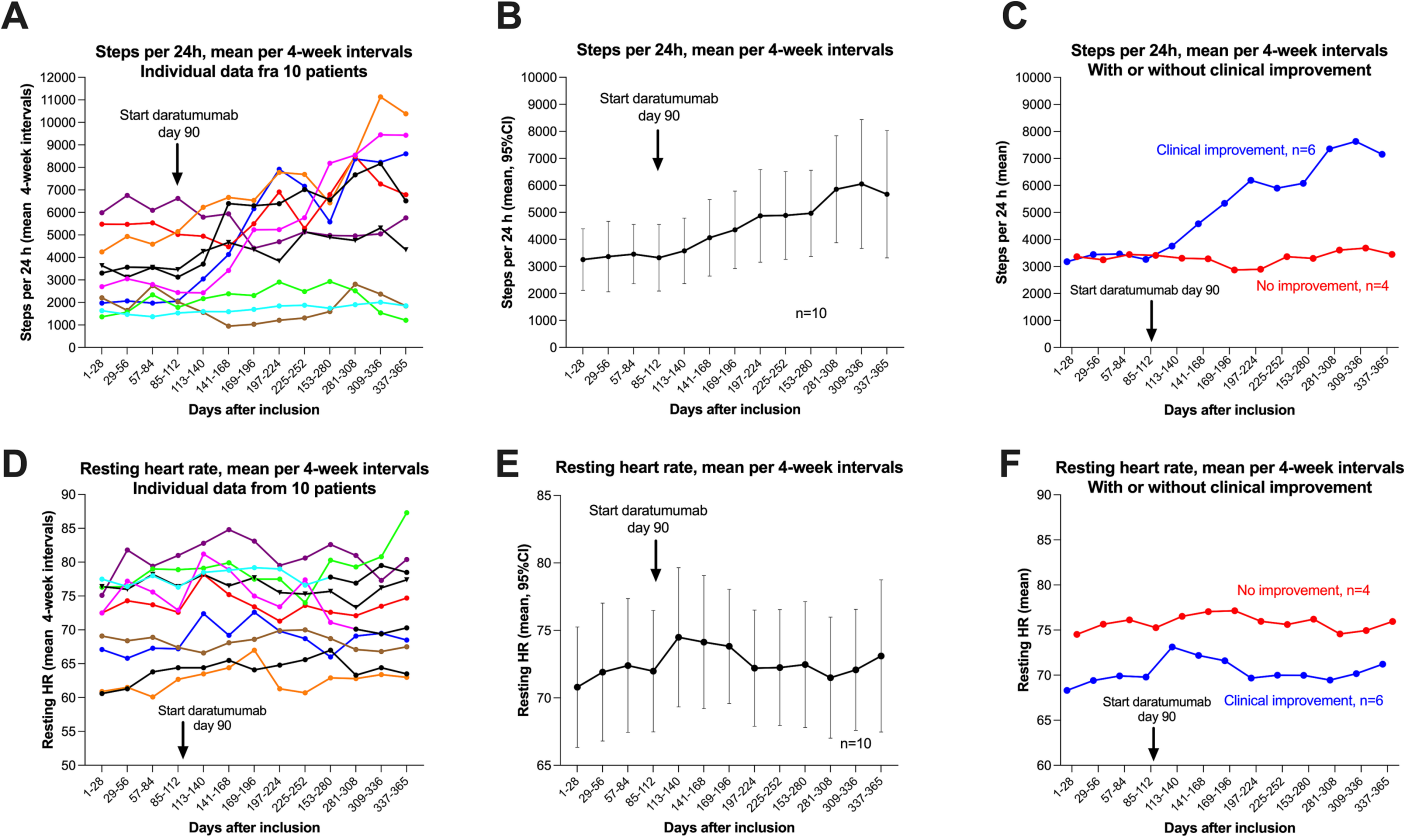
Steps per 24 h and resting heart rate during follow-up. **A–C** show steps per 24 h (means per four-week intervals), with data for 12 months, from week −12 to week 40. Correspondingly, in **(D–F)** are shown the data for resting heart rate (means per four-week intervals). **(A)** (Steps) and D (Resting heart rate) show the raw data with each of the patients represented by a specific colour. **(B)** (Steps) and E (Resting heart rate) show the mean values with 95% CI (per four-week intervals) for the pooled 10 patients. **(C)** (Steps) and **(F)** (Resting heart rate) show the means per four-week interval, divided by patients with clinical improvement (*n* = 6, blue), or with no improvement (*n* = 4, red).

The numbers of steps per 24 h were continuously monitored for 12 months from inclusion by Fitbit Charge activity watch. The mean baseline number of steps per 24 h was 3,359 (range 1,493–6,277), recorded in the time interval −12 to 0 weeks. The baseline number of steps was similar for the group of six patients with later clinical improvement (mean 3,363) and the group of four patients with no improvement (mean 3,353) (Table 1). Figure 5 shows the changes in steps per 24 h through follow-up. Figure 5A shows the mean number of steps per four-week interval for each patient, including the 12 weeks’ run-in period, until 40 weeks after start of intervention. Figure 5B shows the pooled data for 10 patients with means and 95% CI, and in panel C the pooled data divided by six patients with clinical improvement versus four patients with no improvement. Table 2 shows the mean number of steps at 8–9 months. For the patient group as a whole, the mean number of steps increased from 3,359 to 5,862. The corresponding figures for the group of six patients with clinical improvement were 3,363–7,393, and in the four patients with no clinical improvement 3,353–3,566. All five patients with sustained clinical benefit until end of follow-up, recorded single weeks with mean number of steps approximately 10,000 or more (range 9,879–11,949), and single days where they exceeded 15,000 steps.

Figure 5 shows the corresponding data for resting heart rate, for each individual patient (Figure 5D), pooled data for all 10 patients with means and 95% (Figure 5E), and separated in six patients with clinical improvement versus four patients with no improvement (Figure 5F). The baseline mean resting heart rate (run-in period, days 1–84) was 71.7 beats per minute (bpm), and was higher in the group of four patients with no improvement during follow-up (mean 75.8 bpm) versus the six patients experiencing clinical benefit (mean 67.6 bpm). As shown in Figure 5F, the resting heart rate seemingly increased slightly after start of daratumumab injections in the group of six patients with clinical improvement (mean 69.8 bpm 4 weeks before and mean 73.1 bpm the first 4 weeks after start of intervention). In Figure 5D, the green line (patient with known POTS) had a significant increase in resting heart rate, from mean 77 bpm to mean 87 bpm when she experienced a partial relapse toward end of follow-up.

### Adverse events

The primary outcome measures were safety and tolerability as measured by treatment-emergent adverse events, recorded according to CTCAE ver.5.0. Adverse events of grade two or higher are shown in Supplementary Table 1. There were no grade three or four SAE. Generally, the tolerability and feasibility were very good, and all patients complied with the treatment protocol without difficulty.

All patients experienced erythema at the injection site on the abdominal wall, interpreted as AE grade 1. There were no other allergic or injection-related reactions (IRR). Two patients noted slight transient blurring of vision the first few days after daratumumab injection, recorded as AE grade two, which was probably related to study medication.

Most of the patients had one or two episodes of a viral upper airway infection including COVID-19 during 1–2 years’ follow-up, but with an uncomplicated clinical course and no serious clinical features. Two patients with known POTS symptoms had episodes of increased palpitations. One patient experienced palpitations, grade two, after the first injection and again at 22 weeks, and was treated with a short-term increase in her regular dose of metoprolol. The other patient had palpitations for a few hours after each of the first three injections, but not after the fourth to seventh injections. She required no medical treatment. One patient at week 38 had an uncomplicated grade two varicella zoster infection on the thigh, which was treated with oral valacyclovir. One patient with known recurrent bladder infections had several lower urinary tract infections treated with oral antibiotics. There were no other antibiotic courses, and no hospital admissions.

### Immunoglobulin levels in serum and NK cells in peripheral blood

**Figure 6:**
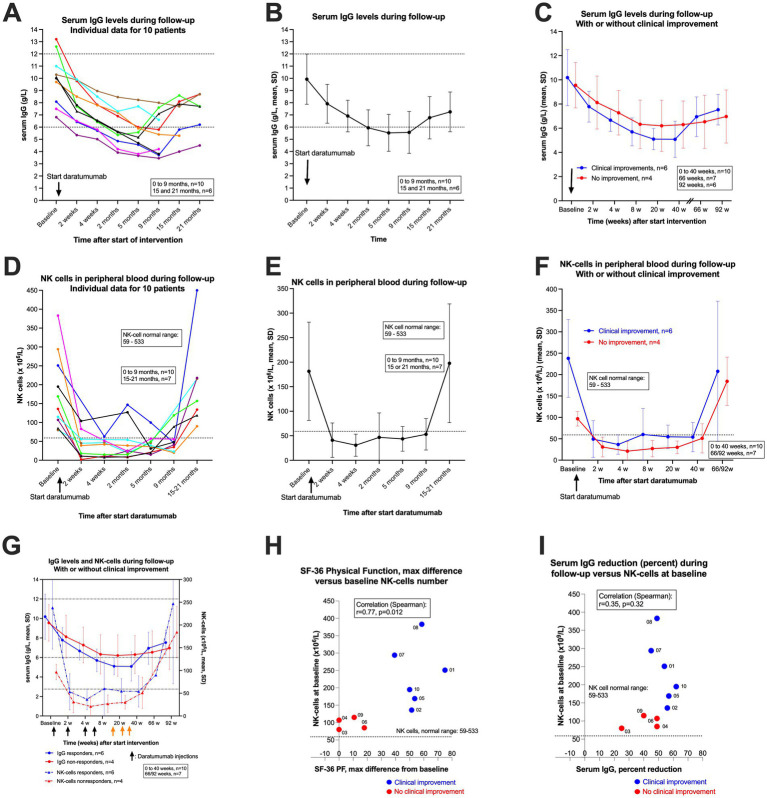
Serum IgG levels and NK cells in peripheral blood during follow-up. **(A–C)** show serum IgG levels during follow-up. In **(A)** individual serum IgG levels for 10 patients (colour codes are identical in the different figures). In **(B)** the means and SD for serum IgG for the pooled 10 patients, and in **(C)** the means and SD for serum IgG levels, divided by patients with clinical improvement (*n* = 6, blue) or with no improvement (*n* = 4, red). The corresponding plots for NK cells in peripheral blood are shown in panel D (individual data for 10 patients), **(E)** (means and SD), and **(F)** (means and SD, with or without clinical improvement). The arrows indicate time points for start of daratumumab injections. **(G)** shows both serum IgG levels and NK cells in peripheral blood during follow-up, with or without clinical improvement. **(H)** shows the correlation between NK-cell numbers at baseline and clinical response expressed as maximum increase in SF-36 Physical Function during follow-up (maximum SF-36 PF minus baseline SF-36 PF; the baseline value for SF-36 PF is the mean of seven recordings from week −12 to week 0). SF-36 PF, scale 0–100, higher value denotes better physical function. **(I)** shows the correlation between baseline NK cells in peripheral blood and maximum relative reduction (in per cent) in serum IgG levels. In **(H,I)** the blue dots represent the six patients with clinical improvement, and the red dots four patients with no improvement during follow-up. The correlation coefficients in **(H,I)** are given by Spearman’s rho.

Table 1 shows the baseline serum immunoglobulin (Ig) levels. Figures 6A–C show serum IgG levels through follow-up until 9 months after start of intervention (*n* = 10) and for the first six patients also with data for 15–21 months. In panel A the individual data for each patient are shown, in Figure 6B the pooled data for all patients with means and SD, and in Figure 6C pooled data divided into the group of six patients with clinical improvement versus four patients with no improvement. The mean baseline serum IgG level was 9.9 g/L (range 6.8–13.2), and during follow-up the lowest mean serum IgG level was 5.5 g/L at 5 months. The corresponding data for the six patients with clinical improvement were mean 10.2 g/L at baseline and 5.1 g/L at 5 months, and for patients with no improvement mean 9.6 g/L at baseline and mean 6.2 g/L at 5 months (Table 2). Thus, the maximum relative reduction of serum IgG level, from baseline during follow-up, was mean 48% in total. In the group of six patients with clinical improvement the mean reduction of serum IgG level was 54%, and among the four patients with no improvement the mean reduction was 40%. As shown in Figures 4, 6, one patient with severe disease (brown line) with no clinical benefit had a maximum relative reduction of serum IgG level of only 22%. Another patient (green line) with a transient moderate clinical improvement followed by partial relapse, had a relatively early increase in serum IgG (Figure 6A).

The IgG subclasses were measured at baseline and at 9 months after start of intervention. The overall relative reduction in IgG1 was mean 44%, in the group with clinical improvement mean 50%, and among patients with no improvement 34% (Table 2). The reduction was evident especially for IgG4 with overall 60% relative reduction; 65% in those with improvement versus 29% among patients with no clinical benefit (Table 2).

However, despite of increasing serum IgG levels toward the end of follow-up (Figures 6A–C), five patients were still in stable remission at 52–104 weeks follow-up. After end of 24 months follow-up, the first patient to receive treatment (blue line) experienced a partial relapse from 26 months after start of intervention, while the second patient (red line) still reports a completely healthy state at 30 months with no ME/CFS symptoms at all.

In Figures 6D–F corresponding data for number of CD16/56 positive NK cells in peripheral blood are shown. As expected from the known CD38 expression on NK cells, 2 weeks after the first daratumumab injection there was a rapid but moderate depletion of NK cells, to a level which in the majority of patients was below the lower limit for the normal range. As shown in Figure 6D, the numbers of NK cells increased slowly during follow-up, especially from 5 to 9 months, and were in the normal range when assessed at 15–21 months.

Interestingly, the baseline numbers of NK cells were in the lower normal range in all four patients with no clinical improvement during follow-up, while the baseline number of NK cells were higher among all six patients experiencing clinical improvement (Figure 6G). There was a significant correlation between baseline number of NK cells in peripheral blood and clinical improvement assessed by maximum increase in SF-36 Physical Function during follow-up, with Spearman’s rho 0.77 (*p* = 0.012) (Figure 6H). The correlation between baseline number of NK cells and maximum relative reduction of serum IgG levels is shown in Figure 6I (Spearman’s rho 0.35, *p* = 0.32). The serum IgA and IgM levels at baseline and through follow-up are shown in Tables 1, 2 and Supplementary Figure 3, with reductions in serum levels as expected after start of intervention, and increasing levels toward end of follow-up, especially among the first six patients who received only the initial four daratumumab injections (the last injection given at week six).

The number of CD4-positive T cells, CD8-positive T cells and CD19-positive B cells, at baseline and during follow-up, are shown in Table 1 and Supplementary Figures 4A1–C2. Generally, there were no systematic changes in number of cells in peripheral blood for these broad subtypes of CD3, CD8, or CD19 positive lymphocytes. Detailed subtyping of lymphocytes by additional CD markers were not performed.

### Autoantibodies and protective antibodies

Screening for the most common clinical autoantibodies revealed positive anti-thyroid peroxidase (anti-TPO) in one patient, with a normal thyroid function. One patient had positive antinuclear antibodies (ANA) with positive anti-CCP IgG and positive ribosomal P IgG. In the other eight patients, there were no positive tests for the common autoantibodies.

Protective antibodies (Diphtheria, Tetanus, SARS-CoV2) were analysed at baseline and after 12 months follow-up. All patients had SARS-CoV-2 antibodies in line with either a previous infection or vaccination. All patients had protective or probably protective levels of antibodies to SARS-CoV-2, Diphtheria and Tetanus, both at baseline and after 12 months.

### Missing data

All patients completed follow-up, with no missing data for the clinical parameters DSQ-SF, SF-36 PF or Function level (%). For steps per 24 h, continuously recorded through 12 months follow-up (weeks −12 to 40) by the Fitbit Charge 5 activity watch, there were complete data for all 365 days in seven out of 10 patients. There were missing data for steps in three patients, for 1, 3, and 20 days, respectively. Thus, there were missing data for steps per 24 h in 24 out of 3,650 days (0.7%).

For resting heart rate, recorded at rest or during the night by Fitbit Charge 5, there were complete data for all 365 days for five patients. There were missing data for resting heart rate in five patients, for 1, 6, 8, 13, and 43 days, respectively. In total there were missing data for resting heart rate for 71 out of 3,650 days (1.9%).

Immunoglobulins were measured at baseline, 2, 4 and 8 weeks, and then 20 and 40 weeks after the first Darzalex injection. For six patients, serum IgG was also measured at 15–21 months after start of intervention. There was one missing value for IgG level, out of 60 measurements (1.7%), and correspondingly for IgA and IgM. There were no missing data for IgG subclass measurements. For CD3, CD4, CD19 lymphocyte subsets, there were in total four out of 60 measurements missing (6.7%). For analyses of protective antibodies at baseline and 12 months (SARS-CoV-2, Diphtheria and Tetanus) there were no missing data. There were no replacements for missing data.

## Discussion

As there are no previous reports on plasma-cell depletion therapy in ME/CFS, the primary objective for this pilot study was to investigate the feasibility and toxicity of subcutaneous daratumumab (Darzalex®) in patients with moderate to severe disease. There were mild adverse events of CTCAE grade 1–2, and no SAE. All patients experienced mild erythema at the injection site, and there were no injection-related reactions (IRR) or allergic reactions. There were, as described above, uncomplicated episodes of lower urinary tract infections in one patient and herpes zoster in another, but no episodes of severe infections and no hospitalization. All patients had episodes of viral upper airway infection, including COVID-19, but uncomplicated. All patients completed the planned daratumumab injections and follow-up.

Six out of 10 patients experienced clinical improvement during follow-up. The pattern of improvement was quite homogenous, starting from 6 to 8 weeks after the first injection, with gradual improvements of all ME/CFS related symptoms through follow-up. During intermittent upper airway viral infections including COVID-19, the improving patients experienced transient symptom worsening, typically of 1–2 weeks’ duration. To us, the improvement or elimination of all ME/CFS symptoms suggests that in this subgroup, central pathomechanistic factors are affected by anti-CD38 intervention. Among the patients with clinical improvement, five out of six experienced pronounced and sustained symptom alleviation until end of follow-up after 12–24 months from inclusion. Thus, in these five, the mean SF-36 Physical Function score at the end of follow-up was 88 (range 80–95) which is close to or within the general population range (44, 45), and the mean maximum increases in SF-36 PF from baseline was 53 (range 39–70) during follow-up. Similar changes were detected through follow-up for DSQ-SF total score and were supported by increases in physical activity level. Toward the end of follow-up, these five patients with sustained clinical improvement described improved health and a major positive impact on their daily lives. One patient with moderate–severe ME/CFS had a transient and moderate clinical improvement, but experienced a gradual relapse toward the end of follow-up (green line in figures). She was among the first six patients included, received four daratumumab injections, and had a relatively early increase in serum IgG levels during follow-up. At extended follow-up at 66 and 92 weeks after start of intervention, she again experienced some improvement in physical functioning, reporting SF-36 PF of 35 at week 66, and SF-36 PF of 50 at week 92, compared to 21.4 at baseline. After the end of 24 months’ follow-up, one patient (blue line) experienced a gradual symptom worsening with partial relapse of ME/CFS, starting from 26 months after inclusion.

The experiences of the six patients with clinical improvement are in sharp contrast to the four patients with no significant clinical impact on the disease course. These had very stable values for SF-36 PF, DSQ-SF total score, Function level and steps per 24 h during follow-up. Thus, except for one transient and moderate response, the data indicate a dichotomous pattern for clinical improvement, either pronounced and sustained through follow-up, or no change at all.

The purpose of daratumumab intervention was to achieve a transient plasma-cell depletion affecting especially the long-lived plasma-cell compartment, with moderate decline in serum IgG levels. The mean relative reduction in serum IgG levels during follow-up was 48% (range 22–62%) and was higher among the six patients experiencing clinical improvement (mean 54%) compared to the four patients with no clinical benefit (mean 40%). At follow-up at 5 and 9 months, the mean serum IgG levels were around the lower level of the normal range, increasing from then on. Interestingly, except for the patient with gradual relapse from 26 months (i.e., after the end of trial follow-up), the gradually increasing serum IgG levels have so far not been accompanied by increasing ME/CFS symptoms. However, longer follow-up will be needed to indicate whether some patients have actually had a lasting effect from transient depletion and subsequent repopulation accompanied by increasing IgG levels, and perhaps an altered balance in the pattern of functional autoantibodies which we believe are important in the pathomechanism.

The IgG subclasses, measured at baseline and at 9 months, showed some differences in the relative reductions during follow-up. The most evident relative reduction of overall 60% was seen for IgG4, with 65% among patients with clinical improvement, versus 29% among patients with no clinical benefit. IgG4 has special characteristics, being the least abundant subclass, with reduced ability to activate Fc-dependent effector functions such as ADCC and complement-mediated cell killing, with stochastic fragment antigen binding (FAB) arm exchange and often bispecific design, although monovalent in antigen binding. IgG4 usually has blocking and inhibitory effects on the target molecule or the immune response, and autoimmune diseases associated with IgG4 autoantibodies are often more responsive to anti-CD20 B-cell depletion intervention (46). Whether the present IgG4 data has pathomechanistic implications for ME/CFS, remains to be elucidated.

As expected, due to their expression of CD38, NK cells in peripheral blood were depleted after the first daratumumab injection, to values either below or close to the lower normal range (59–533 × 10^6^/L) depending on the baseline level. The number of NK cells slowly increased at 5 and 9 months follow-up, depending on whether the patient had the last daratumumab injection at 6 weeks or had additional daratumumab injections until week 30. All patients who completed 15–21 months’ follow-up so far had NK-cell numbers within the normal range.

Interestingly, there was a significant correlation between baseline number of NK cells in peripheral blood and clinical improvement during follow-up, assessed by maximum increase in SF-36 Physical Function, or by maximum decrease in DSQ-SF total score. The four patients without clinical benefit had baseline NK-cell numbers in the lower normal range (mean 97, range 80–115), while those who experienced clinical improvement had higher NK cell numbers at baseline (mean 238, range 136–383). NK cells mediate antibody-dependent cellular cytotoxicity (ADCC) by binding to the IgG Fc region, to ensure adequate plasma-cell depletion when daratumumab binds to CD38. Thus, higher number of baseline NK cells would be expected to enhance plasma-cell depletion, and result in a more marked decline in serum IgG levels. Similarly, one would expect depletion of NK cells when daratumumab binds to CD38 on the NK cells themselves. The patients with no clinical benefit from daratumumab intervention had lower numbers of NK cells in peripheral blood both at baseline and through follow-up until 40 weeks, and also higher serum IgG levels with lower relative reductions from baseline. Whether the observed associations between low baseline NK-cell numbers, serum IgG reductions and clinical improvement during follow-up are biologically determined or coincidental, cannot as yet be determined for certain due to the low number of observations.

According to the literature, in multiple myeloma NK-cell numbers were found to decline with increasing daratumumab exposure, but with little impact on efficacy or safety due to the remaining peripheral blood mononuclear cells (PBMC) fractions maintaining the ability to carry out ADCC, at least ex vivo (47). Also, even though multiple myeloma patients receive more daratumumab injections than in the present study, the NK cells are not completely depleted by daratumumab.

In addition to a reduced number of NK cells in peripheral blood, NK-cell dysfunction in ME/CFS has been described for decades (48). In a recent meta-analysis, reduced NK-cell cytotoxicity was interpreted as the most consistent immune finding in ME/CFS (49), but with high heterogeneity among studies and partly ascribed to different experimental methods. Reduced NK-cell cytotoxicity has also been reported in autoimmune diseases such as systemic lupus erythematosus and rheumatoid arthritis (50). A reduced NK-cell cytotoxicity could perhaps contribute to subtle reactivation of latent infections, such as by herpes virus, thereby maintaining a dysregulated immune response. However, our clinical impression when assessing patients with ME/CFS, is that a subgroup of approximately half of patients report that they have very rarely suffered common viral infections after acquiring ME/CFS, which could rather indicate a hyperactivated innate immune system.

In recent years, several case series have been published demonstrating high efficacy and good tolerability for daratumumab intervention in several treatment-refractory autoimmune diseases, with a role emerging for anti-CD38 antibody therapy (51, 52). In autoimmune haemolytic anaemia (53), anti-phospholipid syndrome (54), treatment-refractory autoimmune cytopenias, cold agglutinin disease, and other autoimmune diseases (ITP, systemic lupus, anti-CASPR2 encephalitis, rheumatoid arthritis, ANCA-associated vasculitis) daratumumab has shown high efficacy in case series (55–57). In systemic lupus erythematosus refractory to B cell depletion, cyclophosphamide and several immunosuppressive drugs, daratumumab was effective and clinical responses sustained by anti-BAFF (belimumab) maintenance treatment (58). These case series reported good tolerability to daratumumab.

A recent review summarized experiences with daratumumab in different refractory autoimmune diseases (59). A total of 38 publications reporting the clinical course of 83 patients were included. The summarized data showed a response rate of remission or improvement in 81%. Among the included studies the median number of daratumumab infusions/injections used was four (range 1–24 injections). Interestingly, the authors found no significant correlation between number of daratumumab doses and either clinical response or hypogammaglobulinemia (59).

Daratumumab is approved for use with chemotherapy and other immune modulatory drugs, or as monotherapy for patients with multiple myeloma or amyloidosis, with formulations for intravenous and subcutaneous administration. The optimal therapeutic daratumumab dosing regimen in ME/CFS is of course not known, and in the present pilot study six patients received four injections, while the next four had in total seven injections. The low number of patients precludes transferable conclusions. However, taking into account the lack of correlation between number of daratumumab injections and clinical response in refractory autoimmune diseases (59), and the observed (possible) association between baseline NK-cell number and clinical improvement during follow-up, one could speculate that the first daratumumab injection may be the most important provided that the NK-cell number at baseline is relatively high.

In a new amendment to the present study, we aim to test this by including four new patients with ME/CFS following a SARS-CoV2 infection who fulfil the trial inclusion criteria, and who have baseline NK-cell numbers above 125 (x10^6^/L). In this amendment, the daratumumab dosing schedule has been reduced. These data will be published later as a case series. Possible interventions for antibody-targeting therapies in Long COVID have recently been summarized (60).

A recent study evaluated the presence of autoantibodies in LC patients with high neurological symptom burden (61), using a human autoantigen microarray, immunoprecipitation, immunofluorescence with patient IgG and a broad range of tissues, and also passive transfer of purified patient IgG to mice that developed neurological symptoms. Diversity of detected autoantibodies was evident (60), and staining seemed to co-localize with pericytes and to some extent with endothelial cells. These data strengthen the case for autoreactivity and functional autoantibodies, induced during the active COVID-19 infection, but persisting and disturbing biological function, as important pathomechanistic factors for neurological symptoms in LC (61).

However, a recent article using platforms for extensive autoantibody screening to extracellular proteins including Rapid Extracellular Antigen Profiling (REAP), reported no significant difference in autoantibody reactivities between ME/CFS patients and healthy controls (17). Lack of reproducibility in reports of autoantibodies in ME/CFS could partly be explained by different techniques for expression of structurally complex proteins, which can make proper autoantibody binding to the epitope problematic.

It is possible that the pattern and balance of functional autoantibodies emerging after systemic infections, which may be agonistic or antagonistic and disturb homeostasis, could be more important than the quantity of specific antibodies. In ME/CFS patients, such patterns of autoantibodies may persist over time with symptoms that to some extent mimic an acute viral infection host response with fatigue, malaise, exercise intolerance, feeling feverish, cognitive symptoms, pain, sore throat, tender lymph nodes and sensory hypersensitivity.

CD38 is an ectoenzyme with multiple and high functional complexity (62). CD38 modulates cell differentiation and signal transduction, and has effector functions during inflammation, including cell recruitment, regulation of NAD availability, and cytokine patterns (63). CD38 is expressed on the cell surface and may also be expressed in intracellular compartments. It is present in many different cell types, but mainly in hematopoietic cells. The highest expression levels are found in plasmablasts, short- and long-lived plasma cells, but also NK cells. Weaker CD38 protein expression is found on subsets of macrophages, B cells and T cells.

Due to the high complexity of the CD38 molecule, the exact mechanisms for the observed beneficial effects using daratumumab in ME/CFS are presently not known. Important functions associated with B-cell subsets could be relevant, such as antigen-presentation and cytokine production (64, 65).

However, we believe our pathomechanistic model, with a role for B cells, plasma cells and functional autoantibodies (12) is supported by the observed clinical responses in a subgroup of ME/CFS patients from daratumumab intervention and concurrent reductions of serum IgG levels, and also taking into account the observed transient effects reported from immunoadsorption experiments (29, 30). In addition, there are several other indications that a variant of an autoimmune mechanism is relevant in ME/CFS, such as a marked female preponderance, associations with autoimmunity-related gene variants (66), and a skewed B-cell receptor gene usage (67). Elderly ME/CFS patients have an increased risk of B-cell lymphoma, especially the low-grade marginal zone lymphomas often associated with autoimmunity or chronic infections (68). Serum B-cell activating factor (BAFF) increase and antigen driven B-cell clonality (69), altered B-cell differentiation (70), and presence of autoantibodies to adrenergic and cholinergic receptors (71) have been reported in ME/CFS. In LC, several studies support an autoimmune pathogenesis (14, 72–74). Although plasma-cell induced depletion by daratumumab with a subsequent decline in autoantibodies was the main goal in this study, we cannot exclude that other induced alterations in the immune balance can be involved in the clinical effects.

The main limitations of this pilot study are the small number of patients, exclusively female participants, and the open label design. Although data are promising, no firm conclusions should be drawn before additional studies have been performed. In June 2025, we launched a randomized, double-blind and placebo-controlled study, including patients with moderate to severe ME/CFS patients according to Canadian consensus criteria, and at least 2 years disease duration. Long COVID patients fulfilling the inclusion criteria may qualify for inclusion.

In this new randomized study, we aim to optimize the design, taking into account our previous experiences from the double-blind study using the anti-CD20 antibody rituximab versus placebo in ME/CFS (37), which came out negative despite promising data from the preceding phase II trials (35, 36). Factors we believe may have influenced that trial outcome, and thus should be evaluated carefully and optimized in a new study, are heterogeneity of the patient sample, natural symptom variation over time interfering with the endpoints especially among patients with milder degree of ME/CFS, outcome measures that proved not stringent enough, recall bias, and placebo mechanisms. Also, although the double-blind design is meant to prevent information bias, uncertainty about group allocation may cause some participants to favor more neutral responses when self-reporting changes over time.

In conclusion, in this pilot study, 10 female ME/CFS patients with high symptom burden and long disease duration received the anti-CD38 antibody daratumumab to target long-lived plasma cells, with subsequent reductions of serum IgG levels. The feasibility was very good with no serious adverse events. Six patients experienced clinical responses, five of whom had major and durable symptom improvements through follow-up. However, definite conclusions should not be drawn before a randomized, placebo-controlled study has been performed.

## Data Availability

The original contributions presented in the study are included in the article/Supplementary material, further inquiries can be directed to the corresponding author.

## Footnotes

^1^ https://dev.fitbit.com/build/reference/web-api/developer-guide/application-design/
