# Correction: Plasma cell targeting with the anti-CD38 antibody daratumumab in myalgic encephalomyelitis/chronic fatigue syndrome—A clinical pilot study

**DOI:** 10.3389/fmed.2025.1677216

**Published:** 2025-08-18

**Authors:** Frontiers Production Office

**Affiliations:** Frontiers Media SA, Lausanne, Switzerland

**Keywords:** ME/CFS clinical trial, daratumumab, autoantibodies, plasma cell, feasibility, pathomechanism

In the Introduction section, paragraph 7, the last sentence incorrectly stated:

However, although specific functional autoantibodies were elevated during acute COVID-19 infection (14), the number and magnitude of autoreactivities to the exoproteome were similar in individuals with or without LC (17), suggesting that the pattern of functional autoantibody responses, rather than single pathogenic autoantibodies, may be relevant.

The correct sentence should read:

However, although specific functional autoantibodies were elevated during acute COVID-19 infection (14), the number and magnitude of autoreactivities to the exoproteome were similar in individuals with or without ME/CFS (17), suggesting that the pattern of functional autoantibody responses, rather than single pathogenic autoantibodies, may be relevant.

The original version of this article has been updated.

