# Supplementary material for: Plasma cell targeting with the anti-CD38 antibody daratumumab in myalgic encephalomyelitis/chronic fatigue syndrome—a clinical pilot study
Source: Front Med (Lausanne). 2025 Jul 9;12:1607353. doi: 10.3389/fmed.2025.1607353 (PMC12283730; doi:10.3389/fmed.2025.1607353)
Supplement: Supplementary file 1 [file Data_Sheet_1.PDF]

Suppl. Table 1. Adverse events during 12 months follow-up, according to CTCAE ver. 5.0.

Patient no.	Term	Grade[a]	Resolved	Relation to study medication
01	External otitis	2	yes	possible
04	Covid-19 infection	2	yes	not related
05	Sinus tachycardia	2	yes	possible
05	Headache	2	yes	possible
05	Lower urinary tract infection	2	yes	possible
07	Covid-19 infection	2	yes	not related
07	Blurred vision	2	yes	possible
08	Blurred vision	2	yes	possible
08	Joint pain	2	yes	possible
08	Palpitations	2	yes	possible
09	Fever after Covid-19 vaccine	2	yes	not related
09	Viral upper airways infection	2	yes	not related
09	Viral upper airways infection	2	yes	unlikely
09	Herpes zoster infection	2	yes	probable
10	Lower urinary tract infection	2	yes	not related
10	Lower urinary tract infection	2	yes	not related
10	Lower urinary tract infection	2	yes	possible
10	Lower urinary tract infection	2	yes	possible
10	Covid-19 infection	2	yes	not related
10	Viral infection	2	yes	unlikely
10	Upper airways infection	2	yes	not related

[a]: According to the protocol, only AE of CTCAE grade 2 or higher have been recorded. There were 21 AE grade 2 during 12 months follow-up, with no AE of grade 3 or higher.
