# Supplementary material for: Plasma cell targeting with the anti-CD38 antibody daratumumab in myalgic encephalomyelitis/chronic fatigue syndrome—a clinical pilot study
Source: Front Med (Lausanne). 2025 Jul 9;12:1607353. doi: 10.3389/fmed.2025.1607353 (PMC12283730; doi:10.3389/fmed.2025.1607353)
Supplement: Supplementary file 2 [file Data_Sheet_2.PDF]

A pilot study using subcutaneous injections of the anti-CD38 antibody daratumumab in 10 patients with moderate to severe Myalgic Encephalomyelitis/Chronic Fatigue Syndrome (ME/CFS)

Resetting the humoral immune response in ME/CFS

Protocol-code: KTS-9-2022

EudraCT no: 2022-000281-18

Sponsor: Haukeland University Hospital, Dept. of Oncology and Medical Physics

Version	Date	Approved	Description of changes in protocol
1.0	20.01.2022		N/A
1.1	28.03.2022		Addition of schedule of activities.<br>A separate risk/benefit assessment section.<br>Details on safety assessments during intervention phase.<br>Details on contraception and pregnancy testing during study.<br>More detailed discussion on daratumumab dosing and administration.<br>Smaller regulatory changes.
1.2	29.04.2022	11.04.2022	New member of Safety Committee
1.3	01.10.2023	17.10.2023	Change in Daratumumab intervention: prolonged interval between treatment 2 and 3, for first two patients.
1.4	23.03.2023	28.04.23	-Inclusion of 4 additional patients, including two with travel distance to Haukeland University Hospital.<br>-Among the 4 new patients, if clinical response at week 24, they will receive 3 additional maintenance daratumumab injections at weeks 28, 34 and 42.<br>-For patients receiving maintenance daratumumab, the follow-up period will be extended with assessments at 60 and 72 weeks.<br>-For registration of any long-term effects from intervention, all 10 patients will have assessments at 18 and 24 months from inclusion.<br>-The total study period is extended to 31.12.2026.<br>-Prophylactic use of Valtrex not given.<br>-New version of patient information and consent form

Følsomhet Intern (gul)

Signatures (place, date, name)

______________________ ______________________ ______________________

______________________ ______________________ ______________________

______________________ ______________________ ______________________

Contents

A pilot study using subcutaneous injections of the anti-CD38 antibody daratumumab in 10 patients with moderate to severe Myalgic Encephalomyelitis/Chronic Fatigue Syndrome (ME/CFS) **1**

Resetting the humoral immune response in ME/CFS **1**

List of abbreviations **5**

Schedule of activities – without maintenance treatment **6**

Schedule of activities – including maintenance treatment **7**

Study centre **10**

List of participants *10*

Background and project description **12**

Myalgic Encephalomyelitis/Chronic Fatigue Syndrome *12*

Previous clinical trials *12*

Pathomechanisms *13*

Plasma cell targeting *16*

Long COVID 17

A pilot study of 10 patients with moderate to severe ME/CFS, using four subcutaneous injections of the anti-CD38 antibody daratumumab, with follow-up for 12 months (KTS-9-2022) **18**

Purpose *18*

Design *18*

End of study 18

Inclusion *18*

Inclusion criteria *18*

Exclusion criteria *19*

Evaluations *19*

Endpoints and statistical analyses *20*

Patient information and informed consent process *20*

Assessment at baseline *21*

Clinical assessment 21

Women of childbearing potential 21

Laboratory tests at baseline *22*

General laboratory tests 22

Immunology 22

Endocrinology 22

Microbiology 22

Blood typing ... 22
Biobank at baseline ... 22

Intervention ... *23*
"Run-in" period before intervention ... 23

Inclusion period ... *23*
Daratumumab intervention ... 23
Daratumumab dosing, injection, and injection-related reactions (IRR) ... 24
Daratumumab dosing and administration ... 25

Drug handling ... *26*

Discontinuation of study intervention and participant discontinuation/withdrawal ... *26*
Study termination ... 26
Discontinuation of study intervention ... 26
Participant discontinuation/withdrawal ... 27

Assessment during follow-up ... *27*

Data registration and management ... *28*

Fitbit charge 4 data ... *29*

Near-Infrared Spectroscopy (NIRS) at baseline and during follow-up ... *29*

Monitoring ... *29*

Biobank samples during follow-up ... *29*

Blood sample collection for the biobank ... *30*
Baseline 0 weeks, and 52 weeks ... 30
At 12, 20, 32 weeks (or at 12, 20, 34, 42 weeks if given maintenance) ... 30

Adverse events and Safety board ... *31*
Adverse events ... 31
Safety board/safety review ... 31

Safety aspects ... *31*

Benefit/ risk assessment ... *32*
Possible benefits ... 32
Possible risks ... 33
Efforts to reduce risks ... 33

Ethical aspects ... *34*

Financial support ... *34*

Publication ... *35*

References ... *35*

Appendix 1: Safety ... 39

Safety monitoring ... *39*

Adverse event/ serious adverse event ... *39*
Definition of adverse events (AE) ... 39
Definition and reporting of serious adverse events (SAE) ... 39

Følsomhet Intern (gul)

Suspected Unexpected Serious Adverse Reactions (SUSAR) 40
Documenting assessment and reporting of adverse events 40
Causality assessment of Serious Adverse Event 42
Follow up of Serious Adverse Events 42

List of abbreviations

ADA	Anti-daratumumab antibodies
ADPR	Adenosine diphosphate ribose
AE	Adverse events
AR	Antibody receptor
BAFF	Serum B-cell activating factor
CADPR	Cyclic adenosine diphosphate ribose
CRPS	Chronic regional pain syndrome
CTCAE	Common terminology criteria for adverse events
DART	Daratumumab as a treatment for adult immune thrombocytopenia (clinical trial)
DSQ-SF	DePaul symptom questionnaire – short form
FcRn	Fc-receptor neonatal
FMD	Flow-mediated dilation
FMD	Flow-mediated dilation
GDPR	General data protection regulation
GLM	General linear model
HADS	Hospital anxiety and depression scale
HLA	Human leukocyte antigen
Ig	Immunoglobulin
IRR	Infusion related reactions
IVIG	Intravenous immunoglobulins
ME/CFS	Myalgic encephalomyelitis/chronic fatigue syndrome
NAD+	Nicotinamide adenine nucleotide
NIRS	Near-infrared spectroscopy
NOMA	Norwegian medicines agency
PEM	Post-exertional malaise
PORH	Post-occlusive reactive hyperemia
POTS	Postural tachycardia syndrome
SAE	Serious adverse events
SF-36	Short Form -36
SF-36 BP	SF-36 Bodily pain subscore
SF-36 PF	SF-36 Physical function subscore
SUSAR	Suspected unexpected serious adverse reactions

Følsomhet Intern (gul)

Schedule of activities – without maintenance treatment

	0	2	4	6	8	10	12	14	16	18	20	22	24	26	28	32	36	40	44	48	52	78	104
**Full clinical assessment [a]**	X						X				X					X					X		
**Telephone consultation**								X [b]	X [b]	X [b]			X [c]		X [c]		X [c]	X [c]	X [c]	X [c]		X [p]	X [p]
**Safety assessment [d]**								X	X	X													
**Registration of adverse events [e]**							X	X	X	X	X	X	X	X	X	X	X	X	X	X	X		
**General laboratory tests**	X [f]						X [g]	X [g]	X [g]	X [g]	X [g]					X [g]					X [g]		
**Serology**	X [f]																						
**Immunology**	X [f]							X [i]	X [i]		X [h,i]					X [i]					X [i,j]		
**Endocrinology**	X [f]																						
**Microbiology**	X [f]																						
**Blood typing/antibody screening**	X [f]																						
**Samples for biobank**	X [k]						X [l]				X [l]					X [l]					X [k]		
**Pregnancy testing (s-HCG)**	X						X		X		X		X [m]		X [m]	X [m]							
**Daratumumab injection**							X	X	X	X													
**SF-36**	X	X	X	X	X	X	X	X	X	X	X	X	X	X	X	X	X	X	X	X	X	X	X
**DSQ-SF**	X	X	X	X	X	X	X	X	X	X	X	X	X	X	X	X	X	X	X	X	X	X	X
**Function level**	X	X	X	X	X	X	X	X	X	X	X	X	X	X	X	X	X	X	X	X	X	X	X
**HADS**	X												X								X		
**Download of Fitbit data**	X	X	X	X	X	X	X	X	X	X	X	X	X	X	X	X	X	X	X	X	X		
**NIRS assessment [n]**	X						X				X									X			

Følsomhet Intern (gul)

Schedule of activities – including maintenance treatment

For patients with symptom improvement after initial 4 injections

	0	2	4	6	8	10	12	14	16	18	20	22	24	26	28	32	34	36	40
**Full clinical assessment [a]**	X						X				X						X		
**Telephone consultation**								X[b]	X[b]	X[b]			X[c]	X[b]	X[c]			X[c]	X[c]
**Safety assessment [d]**								X	X	X				X			X		
**Registration of adverse events [e]**							X	X	X	X	X	X	X	X	X		X	X	X
**General laboratory tests**	X[f]						X[g]	X[g]	X[g]	X[g]	X[g]			X[g,o]			X[g,o]		
**Serology**	X[f]																		
**Immunology**	X[f]							X[i]	X[i]		X[h,i]			X[i]			X[i]		
**Endocrinology**	X[f]																		
**Microbiology**	X[f]																		
**Blood typing/antibody screening**	X[f]																		
**Samples for biobank**	X[k]						X[l]				X[l]						X[l]		
**Pregnancy testing (s-HCG)**	X						X		X		X		X[m]	X	X[m]		X		
**Daratumumab injection**							X	X	X	X				X			X		
**SF-36**	X	X	X	X	X	X	X	X	X	X	X	X	X	X	X	X		X	X
**DSQ-SF**	X	X	X	X	X	X	X	X	X	X	X	X	X	X	X	X		X	X
**Function level**	X	X	X	X	X	X	X	X	X	X	X	X	X	X	X	X		X	X
**HADS**	X												X						
**Download of Fitbit data**	X	X	X	X	X	X	X	X	X	X	X	X	X	X	X	X		X	X
**NIRS assessment [n]**	X						X				X								

Følsomhet Intern (gul)

	42	44	48	52	56	60	64	68	72	78	104
**Full clinical assessment [a]**	X			X							
**Telephone consultation**		X [c]	X [c]			X [c]			X [c]	X[p]	X[p]
**Safety assessment [d]**	X										
**Registration of adverse events [e]**	X	X	X	X		X			X		
**General laboratory tests**	X [g,o]			X [g]							
**Serology**											
**Immunology**	X[i]			X [i,j]							
**Endocrinology**											
**Microbiology**											
**Blood typing/antibody screening**											
**Samples for biobank**	X [l]			X[k]							
**Pregnancy testing (s-HCG)**	X		X[m]	X[m]							
**Daratumumab injection**	X										
**SF-36**		X	X	X	X	X	X	X	X	X	X
**DSQ-SF**		X	X	X	X	X	X	X	X	X	X
**Function level**		X	X	X	X	X	X	X	X	X	X
**HADS**				X							
**Download of Fitbit data**		X	X	X	X	X	X	X	X		
**NIRS assessment [n]**			X								

[a] Full clinical assessment by investigator incl. laboratory tests, AE assessment and laboratory tests

[b] Telephone consultation by study nurse or investigator 2 days prior to treatment. Assessment of AEs and fitness for treatment.

[c] Telephone consultation by study nurse or investigator, monthly after treatment. In the case of AEs of CTCAE grade >1 which require clinical examination and/or extra laboratory workup, visit will be performed in outpatient clinic and not by telephone.

[d] Assessment by study nurse or investigator on day of treatment, prior to injection. Assessment of AEs and fitness for treatment.

[e] Classified by CTCAE version 5.0

[f] See protocol: Laboratory tests at baseline

[g] Hb, TC, DC, TBC, CRP, Na, K, Ca, Mg, P, Glu, Creatinine, eGFR, ALAT, ALP, GT, bilirubin, albumin.

[h] Plasma samples for anti-daratumumab antibodies

[i] Serum immunoglobulins (IgG, IgA, IgM) and lymphocyte subtypes in peripheral blood (CD19, CD3, CD4, CD8, CD56/CD16, ratio CD8/CD4)

[j] IgG subclasses (IgG1, 2, 3 and 4) and vaccination status: titers of antibodies to tetanus, diphtheria, pertussis and Covid-19.

Følsomhet Intern (gul)

[k] 6 ml EDTA plasma, 6 ml EDTA blood, 3x10 ml serum, 2x9 ml Tempus blood RNA
[l] 6 ml EDTA plasma, 2x10 ml serum
[m] On clinical indication only
[n] Near-infrared Spectroscopy during and after limited exercise. Time frames are flexible: Baseline, w12-14, w20-24, w48-52.
[o] Routine blood samples and pregnancy test may be performed within one week before treatment at patient's local hospital or family doctor.
[p] Telephone consultation to register any long-term toxicity and/or symptom change.

Følsomhet Intern (gul)

Study centre

The study will be performed at the Department of Oncology and Medical Physics, Haukeland University Hospital, Bergen, Norway, and will be headed by Prof., consultant in oncology Øystein Fluge and Prof. Olav Mella.

List of participants

Principal investigator, project leader
Prof., consultant in oncology Øystein Fluge
Department of Oncology and Medical Physics,
Haukeland University Hospital
Tel.: 55972010
Mobile: 93044024
E-mail: oystein.fluge@helse-bergen.no
E-mail: oystein.fluge@gmail.com

Investigator, project leader
Prof. Olav Mella
Department of Oncology and Medical Physics,
Haukeland University Hospital,
Tel.: 55972010
Mobile: 90990185
E-mail: olav.mella@helse-bergen.no
E-mail: olav_mella@hotmail.com

Investigator
Consultant in oncology Ingrid Gurvin Rekeland
Department of Oncology and Medical Physics,
Haukeland University Hospital,
Tel.: 55972010
Mobile: 99005697
E-mail: ingrid.gurvin.rekeland@helse-bergen.no
E-mail: ingridgurvin@hotmail.com

Sponsor's representative
Department director, prof. Hans Petter Eikesdal
Department of Oncology and Medical Physics,
Haukeland University Hospital,
Tel.: 55972010

Følsomhet Intern (gul)

Study coordinator
R.N. Kari Sørland
Department of Oncology and Medical Physics,
Haukeland University Hospital,
5021 Bergen
Tel.: 55970439
Mobile: 47719398
E-mail: kari.sorland@helse-bergen.no

Safety board
Prof. Ola Didrik Saugstad
Department of Pediatrics
Oslo University Hospital,
Mobile: 90194144
E-mail: oladsaugstad@hotmail.com

Safety board
Senior consultant in oncology Alexander Fosså
Department of Oncology
The Norwegian Radium Hospital
Ullernchausseen 70
0379 Oslo
Tel.: 22934000
E-mail: aff@ous-hf.no

Translational laboratory work
Prof. Karl J. Tronstad
Institute of Biomedicine
University of Bergen
Tel.: 55586433
E-mail: karl.tronstad@biomed.uib.no

Translational laboratory work
Researcher Ph.D. Ove Bruland
Dept. of Medical Genetics
Haukeland Universitetssykehus
5021 Bergen
Tel.: 55975324
E-mail: ove.bruland@helse-bergen.no

Biobank and laboratory work
M.Sc. Kine Alme
Department of Oncology and Medical Physics,

Følsomhet Intern (gul)

Haukeland University Hospital,
Tel.: 55976255
E-mail: kine.alme@helse-bergen.no

Study monitoring
Section for research and innovation
Haukeland University Hospital

Background and project description

Myalgic Encephalomyelitis/Chronic Fatigue Syndrome

Myalgic Encephalomyelitis/Chronic Fatigue Syndrome (ME/CFS) is a disease of unknown etiology characterized by post-exertional malaise (PEM), sleep disturbances with inadequate restitution, fatigue, pain and sensory hypersensitivity, autonomic dysfunction, cognitive impairment, and several other symptoms. The diagnosis relies on exclusion of other disorders associated with fatigue. There is no validated specific and sensitive biomarker, and no standard approved effective treatment.
Using the Canadian consensus criteria (1), an estimated 0.1-0.8% of the population suffer from ME/CFS (2, 3), which must be distinguished from the much more common general fatigue.
ME/CFS has profound impact on quality of life for patients and their caretakers (4, 5). The socio-economic costs are very high, and there is an urgent need for elucidation of the disease mechanisms, for improved diagnostic approaches, and for rational treatment (6).
ME/CFS often starts in previously healthy individuals after an infection, the most common being infectious mononucleosis (Epstein-Barr virus). It is 3-4 times more frequent in women and influenced by genetic predisposition (7).
The severity varies between patients who are able to participate to some extent in social life (mild), those who are mainly housebound (moderate) or bedridden (severe), and the very severely ill who are completely dependent on assistance for all daily living tasks like feeding or turning around in bed.

Previous clinical trials

Our interest in ME/CFS started in 2007 in our cancer ward, when we observed patients with long-standing ME/CFS who got cancer, and who independently reported that the cancer treatment had beneficial effects on their ME/CFS. The treatments included the cytotoxic drug cyclophosphamide and/or the monoclonal B-cell depleting anti-CD20 antibody rituximab. Our observations led to the working hypothesis that ME/CFS in a subgroup could be a variant of an autoimmune disease, often with a post-infectious onset and with a role for B-cells/plasma cells and antibodies.

Følsomhet Intern (gul)

Based on this hypothesis, we have during the last 12 years tested relevant immunomodulatory drugs in clinical trials for ME/CFS patients. Our clinical trials evaluating the anti-CD20 B-cell depleting antibody rituximab in ME/CFS patients include:

- a pilot case series (8), a small randomized phase II study with a negative primary and positive secondary endpoints (9),
- an open-label phase II study with rituximab maintenance with long-lasting clinical responses in half of patients (10),
- a randomized, multicentre, double-blind and placebo-controlled phase III trial which was negative for the outcome measures (11).

Factors that may have influenced the outcome of the phase III trial include heterogeneity among patients, placebo mechanisms, natural symptom variation, and non-optimal outcome measures.

We also performed an open-label phase II trial using cyclophosphamide intravenous infusions, with clinical responses in half of patients and long-lasting clinical benefit up to four years (12). Cyclophosphamide has broad effects on several subsets of lymphocytes, and the mechanism for benefit in ME/CFS could involve the anti-proliferative effects inhibiting the B-cell activation to plasmablasts. Although there were no severe side effects in our study, cyclophosphamide can induce infertility, and a broader use in ME/CFS patients may be problematic due to toxicity concerns.
Further, we have also seen symptom improvement in ME/CFS patients using bortezomib which mainly targets plasma cells. However, this proteasome inhibitor is also associated with toxicity concerns.

Our pooled experience supports that some ME/CFS patients do respond to rituximab and B-cell depletion, and/or cyclophosphamide. As rituximab targets CD20 positive B-cells, patients whose autoantibody production occurs in CD20 positive (early) plasmablasts would be likely responders. In most patients, however, autoantibodies may be produced in CD20 negative, long-lived plasma cells, which are not targeted by rituximab. This is a known mechanism for lack of response to rituximab in several established autoimmune diseases.

Pathomechanisms

We have recently published an invited viewpoint article in the Journal of Clinical Investigation, focusing on pathomechanisms and possible interventions in ME/CFS (13). In brief, we suggest that ME/CFS is a variant of an autoimmune disease, with a role for B-cells/plasma cells and a pattern of autoantibodies emerging after infection. The alleged pathomechanism and possible targets for intervention are shown in Figure 1 below, copied from our JCI article (13).

Several findings support an autoimmune disease mechanism in ME/CFS. These include a marked female preponderance, a high frequency of autoimmunity among first-degree family members (40-55% in our trials) and enriched HLA risk alleles (14). Furthermore, elderly ME/CFS patients have an increased risk of B-cell lymphoma, especially the low-grade marginal zone lymphomas often associated with autoimmunity or chronic infections (15). Serum B-cell activating factor

Følsomhet Intern (gul)

(BAFF) increase (16), skewed B-cell receptor gene usage (17), and gene expression studies suggesting antigen driven B-cell clonality (18) or altered B-cell differentiation (19), may point to B-cell involvement. In addition, beneficial clinical effects have been reported after immunoadsorption to remove plasma IgG (20).

In established autoimmune diseases, pathogenic IgGs often associate with complement activation, inflammation, and tissue injury. These features are not characteristic of ME/CFS. We suggest that a variant of an autoimmune mechanism affects the autonomic control of blood vessel tone and flow autoregulation. Key symptoms may result from autoantibody-mediated functional disturbance in blood flow autoregulation causing tissue hypoxia on exertion, with secondary autonomic and metabolic responses to maintain energy homeostasis (21). This is associated with lactate accumulation from limited exertion, in some patients even at rest.

There is growing evidence for endothelial dysfunction in ME/CFS affecting large arteries assessed by flow-mediated dilation (FMD), and small arteries assessed by post-occlusive reactive hyperemia (PORH) (22-24). Also, reduced central venous pressure and impaired venous return (“preload failure”) with inadequate cardiac output on exertion, and inadequate oxygen extraction in peripheral tissues due to arteriovenous shunting have been demonstrated (25, 26), possibly due to neurovascular disturbances. These findings could not be explained by deconditioning due to long-lasting disease and inactivity.

Such circulatory disturbances would cause secondary and compensatory adaptations, such as altered autonomic function with increased sympathetic tone. Dysregulated autonomic function has been repeatedly demonstrated in ME/CFS during the last decades (27, 28). A state of inadequate autoregulation of blood flow with hypoxia on exertion would cause secondary metabolic adaptations in efforts to restore energy balance, with increased use of fatty acids and amino acids for fueling and oxidative phosphorylation, also repeatedly shown (21, 29, 30).

Følsomhet Intern (gul)

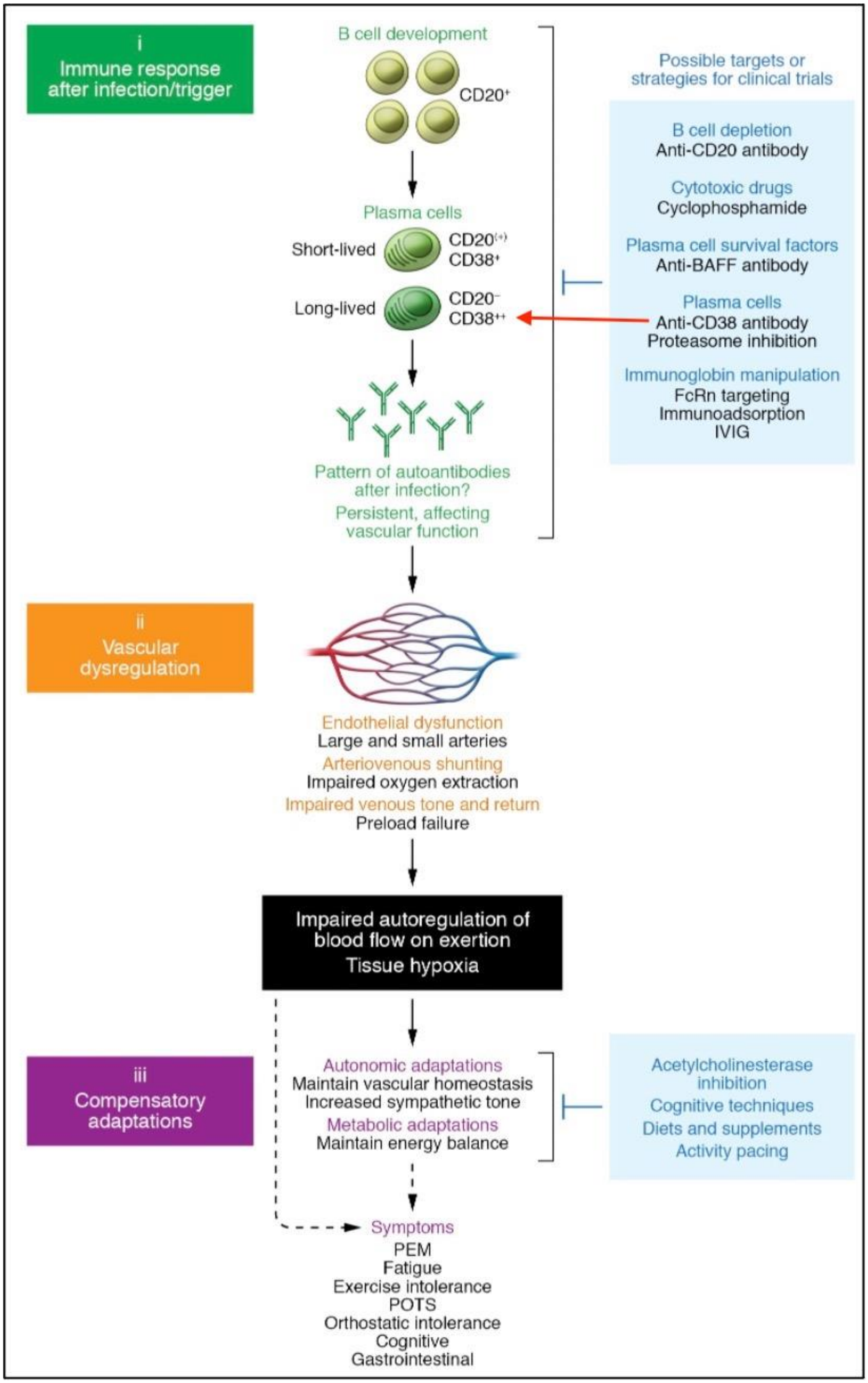

ME/CFS partly overlaps clinically with postural orthostatic tachycardia syndrome (POTS), orthostatic intolerance, fibromyalgia, and complex regional pain syndrome (CRPS). We believe

Følsomhet Intern (gul)

that these illnesses may be associated with a pattern of autoantibodies occurring after triggers such as systemic infections (31), but not resolving properly over time. In ME/CFS, a persistent functional autoantibody pattern could disturb blood vessel autoregulation and lead to secondary metabolic and autonomic adaptations.
To this end, functional G-protein coupled receptor (GPCR) autoantibodies may be involved (32). Agonistic autoantibodies to ß2 adrenergic receptors (AR) and muscarinic 3 receptors have been demonstrated in orthostatic hypotension (33). In POTS, an autoimmune basis has been suggested by the presence of several functional autoantibodies towards GPCRs affecting blood pressure and heart rate regulation, such as antibodies to α1AR, ß1AR and ß2AR (34), and angiotensin II type 1 receptor. Autoantibodies to GPCRs including adrenergic and muscarinic receptors have been investigated in ME/CFS (35). Although GPCR autoantibodies are also present in healthy individuals, it is possible that they contribute in regulatory networks associated with different physiological states and with disease (36).

Thus, we suspect that the autoantibodies in ME/CFS are not complement-activating, nor associated with the histologic inflammation and tissue damage that are seen in many classic autoimmune diseases. Instead, there may be a functional impairment of blood vessel function and autonomic regulation, caused by a pattern of naturally occurring autoantibodies persisting beyond the expected course after the initial infection. Possibly, these naturally occurring autoantibodies are anti-idiotypic (Ab2) and directed against the Fab-fragment of primary antibodies (Ab1) from the infection, and therefore representing "mirror images" of the initial antigen (such as e.g. the spike protein in SARS-CoV2). The anti-idiotypic (Ab2) antibodies may therefore activate or inhibit the cellular structures, and offer an explanation for the presumed natural occurrence with agonist or antagonist activity (37).

Plasma cell targeting

If our interpretations regarding B-cells/plasma cells and autoantibodies in ME/CFS hold true, the production of autoantibodies in a majority of the patients takes place in the mature, long-lived plasma cells. Long-lived plasma cells are refractory to B-cell depletion or to ordinary immunosuppressive drugs (38, 39), and as such, B-cell depletion using rituximab would only be effective as intervention in a minority of patients. Thus, plasma cell targeting and immunoglobulin manipulation emerge as therapeutic opportunities to be explored in approved clinical studies. Possible trials could include drugs that reduce serum IgG by targeting Fc receptor neonatal (FcRn), anti-BAFF antibody targeting B-cell activating factor or anti-CD38 antibodies to target long-lived plasma cells (Figure 1).

Daratumumab is a humanized IgG_{1k} monoclonal antibody directed against the CD38 glycoprotein involved in cell adhesion and signal transduction. The CD38 protein is highly expressed on plasmablasts, short- and long-lived plasma cells, but also with weaker protein expression on subsets of macrophages, B-cells including regulatory B-cells, and T-lymphocytes. CD38 is an ectoenzyme with several functions, which catalyzes the conversion of nicotine adenosine dinucleotide (NAD+) to cyclic adenosine diphosphate ribose (cADPR), and cADPR to ADPR leading to accumulation of adenosine (40).

Følsomhet Intern (gul)

The drug is approved for use with chemotherapy or as monotherapy for patients with multiple myeloma (41, 42). Formulations for intravenous and subcutaneous administration exist.

We have since 2016 discussed the possibility to explore use of the anti-CD38 antibody daratumumab to target autoantibody-producing long-lived plasma cells in ME/CFS. However, although in vitro data have been available, there has been a scarcity of published data evaluating daratumumab in clinical autoimmune diseases. In the last two years, several small case series have been published demonstrating high efficacy and good tolerability in several treatment-refractory autoimmune diseases, and a role for anti-CD38 antibody therapy in systemic autoimmune diseases has been summarized (43).

In autoimmune hemolytic anemia (44), anti-phospholipid syndrome (45), treatment-refractory autoimmune cytopenias, cold agglutinin disease, and other autoimmune diseases (ITP, systemic lupus, anti-CASPR2 encephalitis, rheumatoid arthritis) daratumumab has shown high efficacy in case series (46). In systemic lupus erythematosus refractory to B-cell depletion, cyclophosphamide and several immunosuppressive drugs, daratumumab was effective and clinical responses sustained by anti-BAFF (belimumab) maintenance treatment (47).

In all these case series, the tolerability to daratumumab has been very good, with no severe infusion-related reactions. The patients should however, be monitored carefully for any side effects, including infusion-related symptoms and episodes of neutropenia.
A trial is also ongoing in Norway (DART study) using daratumumab for the treatment of patients with immunological thrombocytopenia (ITP) (Project #2052824 - DART-study - Cristin).

Memory B-cells do not express significant CD38, and re-emergence of antibodies over time would be expected. According to our pathomechanistic hypothesis (13), with autoantibodies emerging after infection which persist and target blood vessel regulation, we believe that ME/CFS disease is a reversible disease. We acknowledge that patients may also experience spontaneous remissions. Elucidation of the mechanisms for symptom maintenance is crucial, but it is conceivable that a "resetting" of the plasma cell compartment could induce a sustained remission in some patients.

Long COVID

Recently, there has been increasing focus on the post-COVID symptoms described as "long COVID", which in many aspects resemble the symptoms of ME/CFS. This applies particularly to patients with relatively mild initial viral disease and no obvious signs of organ damage, but with sustained fatigue, post-exertional malaise and cognitive impairment (48, 49).
Autoantibodies were detected in 50% of COVID-19 patients compared to 15% of controls (50). In addition, a recent study assessed autoantibodies against the complete "exoproteome" (secreted and extracellular proteins) after COVID-19 infection, showing a myriad of autoantibodies with functional impairment of many important immune related and receptor molecules (51).
Manifested long Covid may in time prove to be an entity where immune manipulation can yield benefits.

Følsomhet Intern (gul)

A pilot study of 10 patients with moderate to severe ME/CFS, using four subcutaneous injections of the anti-CD38 antibody daratumumab, with follow-up for 12 months (KTS-9-2022)

Purpose

The main purpose is to evaluate feasibility and safety of daratumumab subcutaneous injections in patients with moderate to severe ME/CFS. We will also collect efficacy data.

Design

An exploratory pilot study of six patients with moderate to severe ME/CFS. After inclusion, there will be an initial 12-weeks observational run-in period, followed by administration of 4 subcutaneous injections of the anti-CD38 antibody daratumumab with 2-week intervals. Patients will be subject to clinical follow-up for 40 weeks from start of intervention.
Four additional patients will receive the same intervention, and if they show improvement of ME/CFS symptoms by clinical evaluation six weeks after the fourth injection (at study week 24), they will receive three additional maintenance daratumumab injections at weeks 26, 34 and 42.

End of study

The study ends when the last included patient has completed their final visit after 52 weeks. For patients with clinical improvement at week 24, who receive additional three daratumumab injections at weeks 26, 34 and 42, the follow-up period will be extended to week 72.

Inclusion

Following approval from the Regional Ethics Committee and the Norwegian Medicine's Agency, we aim to start recruitment in the summer of 2022. The first two included patients will complete 12 weeks run-in and 6 weeks intervention (18 weeks in total). Subject to a safety review, the remaining four patients will then start intervention. End of follow-up of the last patient is planned at the end of 2024.

Participants will be recruited from available lists of ME/CFS patients who have contacted the study center and requested to be evaluated for possible future clinical studies, and may include participants from previous studies. The candidates will be contacted by telephone or in writing, depending on the mode of contact they have themselves specified.

Inclusion criteria

1. ME/CFS according to Canadian consensus criteria (2003) (1);moderate (mainly housebound) to severe (mainly bedridden) disease.
2. Age 18 to 65 years.
3. Signed informed consent.
4. Duration of ME/CFS disease at least two years.
5. The ME/CFS disease should have a defined onset, e.g. after an initial infection or other immunological trigger.

Følsomhet Intern (gul)

6. For females of childbearing potential: Negative serum pregnancy test.

Exclusion criteria

1. Chronic fatigue conditions not fulfilling Canadian consensus criteria.
2. Age under 18 or over 65 years.
3. Mild or mild-to moderate ME/CFS.
4. Very severe ME/CFS, where patient is unable to travel to the hospital for intervention and assessments.
5. Participation in a clinical trial with intervention aimed at ME/CFS during the last two years.
6. Endogenous depression.
7. Known multi-allergy with clinically assessed risk for hypersensitivity to daratumumab.
8. Known contraindication to daratumumab.
9. Significant comorbidity with reduced organ function (kidney, liver, heart, pulmonary).
10. Previous long-term systemic treatment with immunosuppressants the last two years, excluding short steroid courses in e.g. obstructive lung disease.
11. Chronic infections, including chronic hepatitis B or C, HIV, or other relevant infection.
12. Previous or concomitant malignant disease, except basal carcinoma of the skin, or carcinoma in situ in the uterine cervix.
13. Pregnancy or lactation.
14. Inability to comply with protocol including follow-up.

Evaluations

Evaluation of safety and toxicity at 2-week intervals, for 16 weeks after start of intervention (from week 12 to week 28), then at 4-week intervals for the remaining follow-up (from weeks 29 to 52). For patients with clinical improvement at week 24, who receive additional three daratumumab injections, the evaluation of safety and toxicity will be extended to week 72.
This will include registration according to CTCAE ver. 5.0 of adverse events (AE), serious adverse events (SAE), and SUSAR (suspected unexpected serious adverse reactions).

Questionnaires at 2-week intervals during the 12-week run-in period, and during the first 16 weeks after start of intervention, then at 4-week intervals for the remaining follow-up period (to week 52 for patients without maintenance, and to week 72 for patients with three additional maintenance injections).
The questionnaires include:
The Short Form (36) Health Survey (SF36).
DePaul questionnaire Short Form (DSQ-SF).
Function level in percent, according to a form with examples.

In addition, patients will complete the Hospital Anxiety and Depression Scale (HADS) questionnaire at baseline, at 24 weeks, and at 52 weeks.

Fitbit armband data:
Registration of the following variables:

Følsomhet Intern (gul)

Steps per 24 hours.
Resting heart rate.
Heart rate variability.

Mean (and SD) values will be recorded for these variables at 2-week intervals during the 12-weeks run-in period and for 16 weeks from start of intervention (i.e. weeks 0-28), then at 4-week intervals for the remaining follow-up period (from week 29 to week 52).

Endpoints and statistical analyses

The primary outcome measures are safety and tolerability as measured by treatment-emergent adverse events.

The secondary objectives for efficacy are changes in outcome variables, from the baseline/run-in period (12 weeks) before intervention, and through 40 weeks follow-up from start of intervention for SF36 domains including SF36 Physical Function (SF36-PF) and SF36 Bodily pain (SF36-BP), DSQ-SF, Self-reported Function level, and Steps per 24 hours.

The pilot study is explorative, with no blinding or placebo group. We will not describe patients dichotomously as responders or non-responders. Rather, the outcome measures will be descriptive, and assessed by changes in the courses of variables from baseline through 52 weeks from inclusion, including a 12-week run-in before intervention, and 40 weeks after start of intervention. For patients receiving additional maintenance injections at weeks 26, 34 and 42, the analyses will be extended through follow-up to week 72. The outcome variables for efficacy will be assessed by General Linear Model (GLM) Repeated measures for time effects, with the baseline values given as mean values for the 12 week run-in period before start of intervention.

We will also perform analyses for changes in outcome measures at fixed time points compared to baseline, at end of run-in period (i.e. at 12 weeks) and again at 16, 18, 20, 22, 24, 28, 32, 40, 48 and 52 weeks, for patients receiving additional maintenance injections extended at four-week intervals to week 72. Differences in outcome measures from baseline, and from end of run-in at 12 weeks, to the fixed time points after intervention will be assessed by paired t-test or by Wilcoxon paired non-parametric tests.

Extended follow-up may be performed also after end of study, either by clinic attendance or by telephone, to monitor any toxicity or ongoing clinical responses.

Patient information and informed consent process

Candidates will be invited to attend the study centre for a consultation with one of the investigators. This initial visit can be replaced by a telephone consultation.

The candidates will receive a written patient information letter/declaration of consent and given ample time (a minimum of 24 hours) to consider before giving their written consent. No study specific analyses or tests will be performed prior to written informed consent.

Følsomhet Intern (gul)

Participants will also be requested to donate blood samples to the project biobank (2018/1532 «Disease mechanisms in ME/CFS»; Prof. Olav Mella is responsible for the biobank, which is located at the Dept. of Oncology and Medical Physics at Haukeland University Hospital). A separate consent form will be signed before harvesting blood samples for the biobank.

Assessment at baseline

Clinical assessment

One of the investigators will assess the complete disease history for ME/CFS, previous diseases and comorbidity, perform a clinical examination, and will assess any need for further diagnostic evaluation.

The investigator will assess the severity grade of ME/CFS, and may include patients with either moderate (mainly housebound), moderate-to-severe, or severe (mainly bedridden) ME/CFS. The ME/CFS disease onset should be defined and preceded by either an infection or another immunologic stimulus.

If the clinical assessment uncovers other medical conditions which, according to the investigator's assessment, could be the cause of profound fatigue and post-exertional malaise, the patient will not be included.
Conditions that may be interpreted as a cause of persistent profound fatigue include hypothyroidism not adequately substituted with thyroxin, hypocortisolism, malignant disease, chronic infections, chronic pulmonary disease, renal failure, chronic liver disease, chronic heart disease, chronic neurologic disease, endogenous depression, other psychiatric conditions associated with fatigue.

Women of childbearing potential

For women of childbearing potential, a serum pregnancy test at baseline must be negative. A woman is considered of childbearing potential (WOCBP), i.e. fertile, following menarche and until becoming post-menopausal unless permanently sterile. Permanent sterilization methods include hysterectomy, bilateral salpingectomy and bilateral oophorectomy.

A postmenopausal state is defined as no menses for 12 months without an alternative medical cause. Highly effective contraceptive measures include: combined (estrogen and progestogen containing) hormonal contraception associated with inhibition of ovulation; progestogen-only hormonal contraception associated with inhibition of ovulation, intrauterine device (IUD), intrauterine hormone-releasing system (IUS), bilateral tubal occlusion, vasectomized partner, sexual abstinence.

Women of childbearing potential must use effective contraceptive methods for at least 4 weeks before start of treatment and at least 24 weeks following the administration of the last daratumumab injection. A man who is sexually active with a woman of childbearing potential must use a barrier method of birth control for at least 4 weeks before start of treatment and for 24 weeks after intervention.

Følsomhet Intern (gul)

Laboratory tests at baseline

Tests for immunology, endocrinology or serology are not required at baseline if they have been performed in the last 6 months.

General laboratory tests

Hemoglobin, leucocytes with differential count, thrombocytes,
ferritin, s-iron, s-iron binding capacity, transferrin receptor, cobalamin, folate,
Na, K, Ca, Mg, phosphate, glucose, creatinine, urate, total cholesterol, HDL cholesterol, 25-hydroksy vitamin D, ALAT, ALP, GT, bilirubin, CRP, ESR, albumin, total protein, INR.
s-HCG (test for pregnancy) for women of childbearing potential.
Urine analysis (dipstick test).

Immunology

Serum protein electrophoresis, Immunoglobulins IgG, IgG1, 2, 3, 4 subclasses, IgM, IgA.
Lymphocyte subtypes in peripheral blood (CD19, CD3, CD4, CD8, CD56/16, ratio CD8/CD4).
Antibodies to transglutaminase, antinuclear antibodies, anti-CCP, anti-thyroid- antibodies (anti-TPO), antibodies to cardiolipin.
Vaccination status with titers of antibodies to tetanus, diphtheria, pertussis and Covid-19.

Endocrinology

Free-T4, TSH, prolactin, cortisol/ACTH.

Microbiology

Serology for EBV, CMV, Parvovirus B19, Borrelia, HIV, Hepatitis (HBV, HCV).

Blood typing

Daratumumab binds to CD38 is present to some extent also on erythrocytes, which may cause a false positive indirect Coombs test for up to 6 months after the last infusion.
Determination of ABO- and Rh-status will not be affected, while presence of antibodies in serum to weak antigens may be masked.
Blood typing will therefore be performed before start of intervention. The patients will be instructed to carry a card in their wallet describing that they have received daratumumab, with information regarding possible blood transfusions, in case of an emergency.

Biobank at baseline

Blood samples will be harvested for the biobank at baseline:
1 x 6 ml EDTA-Plasma
1 x 6 ml EDTA-Blood
3 x 10 ml Serum
2 x 9 ml Tempus Blood RNA

Følsomhet Intern (gul)

Intervention

After information and signed consent, patients will be instructed on how to fill in the questionnaires at baseline and at intervals during follow-up. The pilot trial set-up is shown in Figure 2.

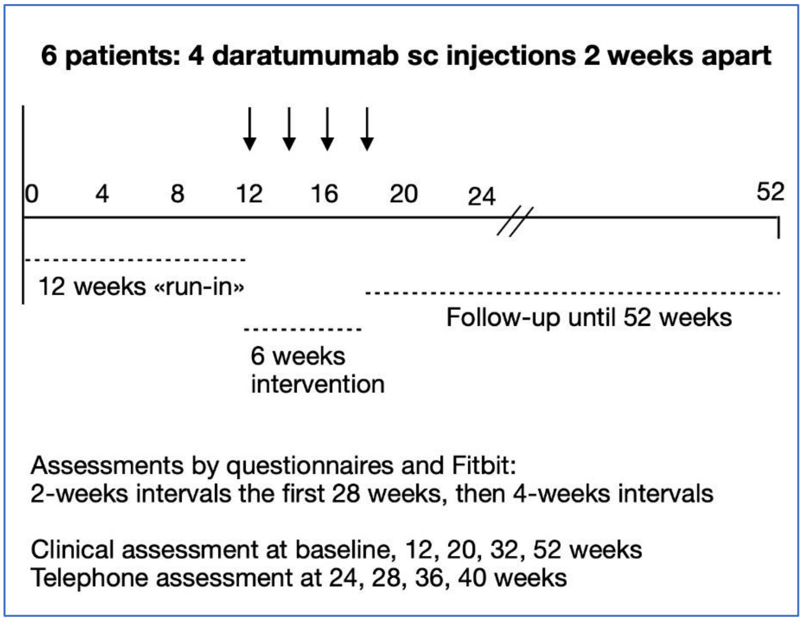

"Run-in" period before intervention

To capture aspects of natural symptom variation over time, patients will receive no intervention during the first 12 weeks of the study, but they will complete questionnaires at 2-week intervals and use the Fitbit armbands for continuous registration of steps per 24 h, resting heart rate and heart rate variability.

Inclusion period

There is limited experience for the use of daratumumab in non-malignant indications. The drug has shown a favorable toxicity profile in the reported small series of patients with diverse autoimmune conditions, as detailed in the project description.
However, daratumumab has not previously been used in ME/CFS. We will therefore start intervention initially in two patients and then wait for at least eight weeks (i.e. at least two weeks after the fourth and last injection) for observation and assessment of any unexpected adverse events, before proceeding to start intervention in the remaining four patients.
The safety board of the study will review and assess safety data after the first two patients have completed intervention.

Daratumumab intervention

After 12 weeks' run-in, patients will start intervention.

Følsomhet Intern (gul)

The first two patients will receive two subcutaneous injections of daratumumab two weeks apart, followed by an observation period of four to six weeks. If there is no serious, unexpected toxicity, we will proceed with another two injections two weeks apart.
After these two patients have received the planned four daratumumab subcutaneous injections, the Safety board will review the data.
Subject to a favorable Safety board review, the pilot study will proceed with the four remaining patients, who will receive four subcutaneous injections of daratumumab two weeks apart.
Thus, all patients will receive in total four subcutaneous injections of daratumumab.
According to a second amendment to the protocol, among four additional patients included, provided clinical improvement at week 24, these will receive additional three daratumumab injections, at weeks 26, 34 and 42, and with extended follow-up until week 72.

The injections will be administered in the outpatient clinic in the Department of Oncology and Medical Physics at Haukeland University Hospital.

Oral premedication will be given at least one hour before daratumumab injection. Before the first daratumumab injection: Dexamethasone 10 mg orally, Cetirizine 10 mg orally, Paracetamol 1 g orally, Montelukast 10 mg orally. Also, at least 20 minutes before daratumumab: Dexchlorpheniramine 5 mg iv.
On the following two days (2 and 3): Dexamethasone 4 mg orally.

Before the second and subsequent daratumumab injections, if no reaction to the first injection: Dexamethasone 10 mg orally, Cetirizine 10 mg orally, Paracetamol 1 g orally.

Daratumumab dosing, injection, and injection-related reactions (IRR)

Daratumumab subcutaneous injections will be given at a fixed dose of 1800 mg. A 15 ml syringe, which also contains recombinant hyaluronidase, is injected over 3-5 minutes into the abdomen approximately 7-8 cm to the right or left of the umbilicus (rotating injection sites for successive injections), on intact, healthy skin.

The patient will stay in the outpatient ward for at least 6 hours after the first Daratumumab injection, for observation of any injection-related reactions (IRR). Intravenous access will be established before the injection for administration of premedication and will be maintained throughout the observation period. Any allergic reactions or IRR will be treated according to standard hospital procedure.
If no IRR after the first injection, the observation period following the subsequent three injections will be 2 hours.

IRR after subcutaneous injections occurred in 9% of patients with multiple myeloma after the first injection, usually of grade 1-2. The risk of IRR will probably be lower in these patients without malignant plasma cells due to the low number of CD38 positive plasma cells. In the small patient series using daratumumab in autoimmune diseases, IRR was not reported to be a clinical problem.

Følsomhet Intern (gul)

If a patient suffers a severe allergic reaction or injection-related reaction (grade 4), the patient will not receive further daratumumab intervention.

Daratumumab dosing and administration

The anti-CD38 antibody daratumumab has not been tested previously in ME/CFS patients. During recent years, small phase II studies have been published evaluating daratumumab in several autoimmune diseases, among them patients refractory to treatment including rituximab, cyclophosphamide and other immunomodulatory drugs.
In autoimmune hemolytic anemia (44), anti-phospholipid syndrome (45), treatment-refractory autoimmune cytopenias, cold agglutinin disease and other autoimmune diseases (ITP, systemic lupus, anti-CASPR2 encephalitis, rheumatoid arthritis), daratumumab has shown high efficacy in case series (46). In systemic lupus erythematosus, daratumumab was effective and clinical responses sustained by anti-BAFF (belimumab) maintenance treatment (47).
The use of daratumumab in ME/CFS is based on the pathomechanistic model suggesting that ME/CFS is a variant of an autoimmune disease with a role for autoantibodies, produced by long-lived plasma cells.

It is presently unknown what would be the optimal administration and dosing schedule in ME/CFS. Subcutaneous daratumumab 1800 mg is in multiple myeloma equivalent to intravenous daratumumab 16 mg/kg. Subcutaneous daratumumab in monotherapy has a half-life of 20.4 days.
We have based the suggested dosing schedule on the experiences from the above-mentioned studies in established autoimmune diseases. In these small case series of patients with warm-type autoimmune hemolytic anemia (wAIHA), the daratumumab administrations span from one, two, four, six and more than ten administrations, summarized in (46).
In one study of four patients with autoimmune hemolytic anemia (44), patients received repeated daratumumab infusions up to a maximum of six doses, including one patient who stopped treatment after three administrations due to a very good response, with lasting responses to end of follow-up after one year in two patients, and with no major side effects.

In other autoimmune diseases, pilot case reports have used four doses (immune-mediated thrombocytopenia after allogeneic stem cell transplantation; anti-phospholipid syndrome), four daratumumab doses (systemic lupus erythematosus) followed by anti-BAFF antibody belimumab maintenance, 10 administrations (anti-NMDA encephalitis) and 13 administrations (anti-CASPR2 encephalitis), summarized in (46).

Memory B-cells do not express significant amounts of CD38, and re-emergence of antibodies over time would be expected. According to our pathomechanistic hypothesis (13), with autoantibodies emerging after infection which persist and target blood vessel flow regulation, we believe that the ME/CFS disease is a reversible disease. We acknowledge that patients may also experience spontaneous remissions. We do not know if and when the putative autoantibodies will reappear after daratumumab intervention, but it is conceivable that a "resetting" of the plasma cell compartment could induce a sustained remission in some patients.

Følsomhet Intern (gul)

In this pilot study, we have in the original protocol not included any maintenance treatment to prolong possible clinical responses. We anticipate that four administrations of standard daratumumab dosing in each injection will be sufficient to assess feasibility and toxicity, and also to give indications for possible clinical benefit.

Based on observations of patients in the pilot study until March 2023, according to a second amendment, four additional patients will be included in the pilot study. For these four new patients, if clinically assessed improvement of ME/CFS symptoms at week 24, these will receive three additional daratumumab injections 8 weeks apart, i.e. at weeks 26, 34 and 42, and with prolonged follow-up until week 72.
A possible future larger study could verify or refute such efficacy, and if we observe clinical responses with limited duration after daratumumab, possible options include a prolonged daratumumab induction treatment or inclusion of a maintenance phase using e.g. B-cell depletion with rituximab, or anti-BAFF antibody treatment with belimumab.

Drug handling

The pilot study is an open-label phase II study with no blinding or placebo group. Daratumumab will be available from the hospital pharmacy. Records of prescription and administration of daratumumab will be maintained through the medication management software Cytodose, as per standard department procedures.

Daratumumab must not be utilized after the expiry date printed on the label. Daratumumab must be protected from light and must not be frozen. When taken from storage at 2-8°C, the syringe will be used within 2 hours, and the vial must not be put back in the refrigerator. The product does not contain preservatives, therefore, any unused portion remaining in the vial must be discarded.

Discontinuation of study intervention and participant discontinuation/withdrawal

Study termination

If any new information affecting the risk-benefit assessment should emerge, and upon the request of investigators or the Safety Board, sponsor will suspend the study pending a new risk-benefit assessment. If the new information indicates that the patient risks would outweigh the benefits, the study will be terminated.

If the study is prematurely terminated or suspended, the sponsor shall promptly inform the investigators, the regulatory authorities and the participants.

Discontinuation of study intervention

In rare instances, it may be necessary for a participant to permanently discontinue study intervention. If study intervention is permanently discontinued, the participant should, if at all

Følsomhet Intern (gul)

possible, remain in the study to be evaluated for safety and efficacy as described under "Assessment during follow-up".

A participant may be withdrawn at any time at the discretion of the investigator for safety reasons, if the investigator considers that further intervention may present a danger to the patient's health. If a patient suffers a severe allergic reaction or injection-related reaction (grade 4), the patient will not receive further daratumumab intervention.

Participant discontinuation/withdrawal

A participant may withdraw from the study at any time at the participant's own request for any reason (or without providing any reason).
If the participant withdraws consent for disclosure of future information, the sponsor may retain and continue to use any data collected before such a withdrawal of consent.
If a participant withdraws from the study, the participant may request destruction of any samples taken and not tested, and the investigator must document this in the site study records.

Assessment during follow-up

After inclusion, patients will complete the questionnaires (SF36, DSQ-SF, self-reported Function level) at 2-week intervals for the first 28 weeks, then at 4-week intervals for the remaining 24 weeks (until 52 weeks). For the additional four included patients (according to amendment 2), if clinically assessed improvement at week 24, there will receive additional three daratumumab injections as described, and with prolonged follow-up until week 72.
Steps per 24h, Resting heart rate, and Heart rate variability will be registered by mean (and SD) per 2-week intervals for the first 28 weeks, then at 4-weeks intervals for the remaining 24 weeks (or 44 weeks if given maintenance) of the study.

Patients will be assessed at the outpatient clinic, Dept. of Oncology, at 12 weeks (end of run-in period before start of daratumumab intervention) and at 20 weeks (after the fourth daratumumab injection), 32 weeks and at end-of-study at 52 weeks.

At these visits (12, 20, 32, and 52 weeks) a clinical assessment with laboratory test will be performed, in addition to collection of self-report questionnaires and blood samples for biobank.
Patients will also be assessed by clinical examination and laboratory tests before each daratumumab injection at 14, 16 and 18 weeks. An investigator or study nurse will contact the patient by telephone 2 days before daratumumab injections number 2, 3 and 4, and the physician will assess the patient upon arrival at the outpatient clinic for planned daratumumab injections 2, 3 and 4.
The study nurse will record any complaints or side effects from intervention, and the investigators will assess the CTCAE grading.
To make participation in the pilot study less demanding for patients, further monthly assessments at 24, 28, 36, 40 and 44 weeks can be performed by telephone provided that the

Følsomhet Intern (gul)

patients experience no significant side effects. However, if the patient experiences significant side effects – i.e. side effects of CTCAE grade > 1, which require clinical examination and/or extra laboratory work-up, these visits should also be performed in the outpatient clinic.

The patients should use effective contraception measures from 4 weeks before start of intervention and until 24 weeks after last intervention (i.e. week 44).
In the intervention period (weeks 12-18) and for the following 14 weeks (i.e. until week 32) female patients of child-bearing potential must use highly effective contraceptive measures, and in this time interval the patients must perform a monthly serum pregnancy test (weeks 12, 16 and 20). Thereafter, in the time interval 24-32 weeks, pregnancy tests can be taken according to clinical indication.

At 12, 20, 32 and 52 weeks:
Laboratory tests: Hemoglobin, leucocytes with differential count, thrombocytes, CRP, Na, K, Ca, Mg, phosphate, glucose, creatinine, eGFR, ALAT, ALP, GT, bilirubin, albumin.
At these visits, blood samples for biobank will be collected.

At 14, 16 and 18 weeks (before daratumumab injections), and at weeks 26, 34 and 42 if given daratumumab maintenance:
Laboratory tests: Hemoglobin, leucocytes with differential count, thrombocytes, CRP, Na, K, Ca, Mg, phosphate, glucose, creatinine, eGFR, ALAT, ALP, GT, bilirubin, albumin.

In addition:
At the **20-week visit**, plasma samples for determination of anti-daratumumab antibodies will be collected.

At the visits at **12, 16 and 20 weeks, and if given maintenance at weeks 26, 34 and 42:** s-HCG.

At the visits at **weeks 14, 16, 20, 32 and 52, and if given maintenance at weeks 26, 34 and 42**: serum immunoglobulins (IgG, IgA, IgM) and lymphocyte subtypes in peripheral blood (CD19, CD3, CD4, CD8, CD56/16, ratio CD8/CD4).

Also at the **52 week visit**: IgG subclasses (IgG1, 2, 3 and 4), and vaccination status with titers of antibodies to tetanus, diphtheria, pertussis and Covid-19.

Data registration and management

The recorded questionnaires (SF36, DSQ-SF, Function level), the weekly downloaded Fitbit data (steps per 24 hours, resting heart rate, heart rate variability) and any adverse events of CTCAE grade 2 or higher, will be registered in the CRF.

Følsomhet Intern (gul)

Fitbit charge 4 data

Patients will receive a Fitbit Charge 4 armband at study start, with detailed instructions. The patients will be instructed to use Fitbit continuously except when recharging once weekly. Fitbit activity data from each participant will be downloaded at the study centre weekly, using the Fitbit web API (https://dev.fitbit.com/build/reference/web-api/developer-guide/application-design/)

After our randomized, placebo-controlled and double-blind trial with rituximab proved negative (11), we have worked to improve the design of future intervention trials in ME/CFS. We recently performed an observational study of 27 ME/CFS patients over 6 months, without intervention, using QoL and disease-specific questionnaires as well as continuous monitoring of steps and resting heart rate.

We will use the set-up from the Fitbit activity study (as yet unpublished). The study was approved by the Regional Committees for Medical and Health Research Ethics in Norway (REK No. 28780). A Data Protection Impact Assessment was performed in consultation with the IT Security Manager and Data Protection Officer for the Bergen Hospital Trust and user representatives.

Fitbit's terms of use comply with the General Data Protection Regulation (GDPR) directive. In order to protect the participants' privacy, we will use pseudonymisation toward third parties. Each participant Fitbit account will be set up using a study-specific e-mail address, initials instead of name and a fictitious date of birth.

Near-Infrared Spectroscopy (NIRS) at baseline and during follow-up

We are currently in the process of testing a method for non-invasive measurements of tissue oxygenation using Near-Infrared Spectroscopy (NIRS) technology. NIRS measurements may be performed at baseline and repeated in the intervals 12-14 weeks, 20-24 weeks and 48-52 weeks. NIRS measures in real time the relative oxyhemoglobin and deoxyhemoglobin concentrations in the tissue at a distance approximately 1,5 cm below the probe, and provides an estimate of tissue oxygenation. We will measure tissue oxygenation at rest, continuously through a standardized repeated exercise, and during recovery. The exercise lasts for one minute followed by two minutes rest and is repeated twice. Volunteers who have tested the method have tolerated this limited exercise well.

Monitoring

The pilot study will be monitored by the Section for research and innovation at Haukeland University Hospital

Biobank samples during follow-up

At 12, 20, 32 weeks:
If receiving additional maintenance injections: at weeks 12, 20, 34, 42:
1 x 6 ml EDTA-Plasma
2 x 10 ml Serum

At 52 weeks

Følsomhet Intern (gul)

1 x 6 ml EDTA-Plasma
1 x 6 ml EDTA-Blood
3 x 10 ml Serum
2 x 9 ml Tempus Blood RNA

Blood sample collection for the biobank

The biobank form is marked with personal ID and patient study ID. Corresponding vacutainers are marked with patient study ID only.
Sample collection are performed in the following order:
Serum, BD Vacutainer Clot Activator Tube, red top
Plasma, Vacuette K2EDTA, purple top
Blood, Vacuette K2EDTA, purple top
Blood, RNA, Applied Biosystems TEMPUS Blood RNA Tube, blue top

Serum and EDTA-tubes are turned upside down 10 times after blood collection. Tempus tubes must be shaken vigorously for at least 20 seconds after blood collection, until a black color appears.

Baseline 0 weeks, and 52 weeks

3x10 ml serum, BD Vacutainer Clot Activator Tube, red top
- Let coagulate for minimum 30 minutes, to avoid hemolysis during spinning.
- Spin at 1500xg, 10 minutes at room temperature.
- aliquot serum, 0,5 mL, on ice

1x6 ml Plasma, Vacuette K2EDTA, purple top
- Leave for maximum 1 hour at room temperature.
- Spin at 1500xg, 10 minutes, at room temperature.
- aliquot plasma, 0,5 mL, on ice.

1x6 ml Blood, Vacuette K2EDTA, purple top
2x9 ml Blood, RNA, Applied Biosystems TEMPUS Blood RNA Tube, blue top
All Serum-aliquots, plasma-aliquots, EDTA-blood (6 mL) and TEMPUS Blood RNA-tubes are stored at -80°C.

At 12, 20, 32 weeks (or at 12, 20, 34, 42 weeks if given maintenance)

2x10 ml serum, BD Vacutainer Clot Activator Tube, red top
-Let coagulate for minimum 30 minutes, to avoid hemolysis during spinning.
- Spin at 1500xg, 10 minutes at room temperature.
- Aliquot serum, 0,5 mL, on ice

1x6 ml Plasma, Vacuette K2EDTA, purple top
- Leave for maximum 1 hour at room temperature.
- Spin at 1500xg, 10 minutes at room temperature.
- aliquot plasma, 0,5 mL, on ice.

Serum-aliquots and plasma-aliquots are stored at -80°C.

Følsomhet Intern (gul)

Adverse events and Safety board

Adverse events

Investigators will assess and classify adverse events (AE), serious adverse events (SAE), and SUSAR (suspected unexpected serious adverse reactions) according to the Common terminology criteria for adverse events (CTCAE), version 5.0 and the study drug SmPC.

For the purpose of safety and toxicity assessment, adverse events from grade 2 (moderate) and upwards, with possible, probable or certain causal relationship with the study drug will be reported. All SAE will be reported, regardless of causality.

Elective or pre-planned hospital admissions arranged prior to the start of study, or planned hospital admissions for trial specific procedures or treatment are not considered SAE.

Definitions and procedures for assessments and reporting of adverse events are described in appendix 1.

Safety board/safety review

The safety aspect of the study will be monitored by Prof. Olav Dahl, Haukeland University Hospital and Dr. Alexander Fosså, Dept. of Oncology, The Norwegian Radium Hospital, Oslo University Hospital. Members of the Safety board will be independent of the study investigators. They will monitor registered adverse events (AE) and serious adverse events (SAE), but will not perform clinical examinations of the patients.
The first safety review will be performed after the first two participants have completed all four daratumumab injections (at week 18-20), and before start of intervention for the remaining four patients. There will also be a safety review after all six patients have completed intervention.

Safety aspects

The published case series using daratumumab in autoimmune diseases have reported good tolerability. The relatively high frequency of **infusion-related reactions (IRR)** seen in multiple myeloma, especially at first infusion, has not been observed as a clinical problem in the small published series of autoimmune conditions, possibly due to the low number of plasma cells in bone marrow and tissue niches compared to multiple myeloma patients who often carry a high tumor burden.
Moreover, aggravation of neutropenia and thrombocytopenia, which has been a concern during multiple myeloma treatment with daratumumab and concomitant chemotherapy, has not been reported in the case series of daratumumab monotherapy in autoimmune diseases.

Hypogammaglobulinemia from repeated anti-CD38 antibody intervention and plasma cell depletion may be a matter of concern, especially with long-term treatment. We do not anticipate that this will be a clinical problem when administering a mere four injections, but we will assess immunoglobulins regularly through follow-up, and also measure IgG subclasses at baseline and at the end of follow-up.

Følsomhet Intern (gul)

We will assess vaccination status with titers of antibodies to tetanus, diphtheria, pertussis and Covid-19, at baseline and repeated at end of follow-up (52 weeks).

Because daratumumab has not been used for ME/CFS, we will assess any occurrence of **anti-daratumumab antibodies (ADA)**, in plasma samples 2 weeks after the fourth injection (at week 20). Plasma samples will be screened for antibodies binding to daratumumab and the titer of any confirmed positive samples will be reported.

The efficacy and safety of the anti-CD38 antibody daratumumab in ME/CFS is unknown. We do not know whether the drug will be beneficial. In addition to high expression of plasmablasts and long-lived plasma cells, CD38 is also expressed on diverse subsets of B- and T-lymphocytes, including B-regulatory cells. Therefore, all biological and clinical effects may not be foreseen.

Although the tolerability in known autoimmune diseases, assessed from the small case series published so far, has been good, we do not know if there will be other side effects in ME/CFS. In theory, some patients could experience worsening of the disease. With this in mind, we will perform a safety review after treatment of the first two patients as described under "Intervention".

Benefit/ risk assessment

Patients with moderate (mainly housebound) or severe (mainly bedridden) ME/CFS suffer a very high symptom burden, where even minimal physical or mental activity can trigger a major aggravation and post-exertional malaise. Comparing patients with different chronic diseases, several studies have shown that ME/CFS patients report the highest levels of symptom burden (4, 5). Patients are often cut off from social relations, work or education, and there are major implications including financial difficulties for both patients and caregivers.

The health system has little to offer these patients, and there is a general lack of recognition of the disease, which is largely due to the lack of scientifically approved pathomechanisms, objective biomarkers, and standard effective treatments. Due to the lack of uniformly accepted disease mechanisms, there have been very few clinical intervention studies. Few research groups worldwide have performed interventional studies using immunomodulatory drugs in ME/CFS. This is in sharp contrast to the magnitude of the ME/CFS problem in society, affecting 0.1-0.8% of the population. Thus, there is an urgent need for clinical intervention trials based on scientifically sound hypotheses.

Possible benefits

The participants will gain access to a new and explorative treatment for ME/CFS, and may experience a beneficial effect on the disease course and symptoms. The patients will undergo a thorough clinical assessment before inclusion and close follow-up for the 12-month study period. After end of study, we will continue to monitor any ongoing clinical response or side effects.

Følsomhet Intern (gul)

The pilot study will give information of feasibility and possible toxicity, and can also indicate any possible beneficial effects for ME/CFS patients. Such information is necessary to plan a future larger study among ME/CFS patients aiming to verify or refute clinical efficacy.
If daratumumab intervention were to show a beneficial clinical effect on ME/CFS symptoms and disease course, this could be of great importance for a large patient group estimated to 15 to 20 million worldwide. Trial data may also pave the way for elucidation of disease mechanisms, which may form a basis for further therapeutic strategies and for recognition of the ME/CFS disease in medicine as well as in society.

Possible risks

The participants may experience side effects from the intervention. We cannot be certain that the anti-CD38 antibody treatment will be associated with a beneficial disease course, and patients could in theory also experience worsening of their ME/CFS symptoms.
A lack of clinical response may represent a psychological burden to the patients.
For patients with moderate (mainly house-bound) or severe (mainly bedridden) ME/CFS, participating in a clinical trial may cause temporary worsening due to the stress involved in performing study-specific procedures, such as travelling to the hospital, taking blood samples, receiving subcutaneous interventions and completing questionnaires.

Efforts to reduce risks

Our research group, based in an oncology department, has access to the necessary treatment and laboratory facilities, and we have clinical experience with follow-up of ME/CFS patients as well as extensive experience with immunomodulatory drugs. We will make efforts to reduce the stress and burden for patients participating in the pilot study, with facilitation for treatment in the outpatient cancer ward. The patients will have direct access to the study management throughout the study period, and will also be able to contact the doctor on duty at the Dept. of Oncology, Haukeland University Hospital if and when necessary.
The participants will undergo regular clinical and laboratory assessments as outlined in the protocol. They will be observed in the outpatient clinic for 6 hours after the first injection, and at least 2 hours after the remaining 3 injections. They will receive pre-medication before daratumumab injections to minimize the possibility for injection-related reactions. Measures will be taken to prevent complications such as virus reactivation and interference with pre-transfusion screening, as outlined under *Intervention* and *Blood typing*.

The first two patients will receive two subcutaneous injections of daratumumab two weeks apart, followed by an observation period of four to six weeks. If there is no serious, unexpected toxicity, we will proceed with another two injections two weeks apart.
After these two patients have received the planned four injections of daratumumab, and before the remaining four patients start intervention, an independent Safety board will perform a formal assessment on safety.

We have since 2016 discussed the concept of plasma cell targeting using the monoclonal anti-CD38 antibody daratumumab ME/CFS, but have delayed an application because there has been limited published data on the use in non-malignant indications. However, the general

Følsomhet Intern (gul)

impression from recent studies using daratumumab in several autoimmune disease, is that the drug is well tolerated with a favorable toxicity profile, and with good efficacy in antibody-mediated diseases in which the autoantibodies are produced by long-lived plasma cells. There have been few infusion-related reactions, which are relatively often seen in multiple myeloma.

This pilot study is explorative, aiming primarily to assess feasibility and possible toxicity, and to indicate possible beneficial effects. The low number of participants prevents firm conclusions for efficacy, but may provide the rationale for performing a larger clinical trial.
Considering the quality of life for patients with moderate to severe ME/CFS, we believe that assessment of the anti-CD38 treatment principle in a small pilot study for evaluation of safety and possible effect is both justified and important.

Ethical aspects

See Benefit/risk assessment for a detailed discussion of possible benefits and risks to the patient.

The pilot study will be performed according to the approved protocol, applicable laws and regulations and the ICH Good Clinical Practice (GCP) guideline.

Participation in the trial will be subject to written informed consent, and all participants will be informed of their right to withdraw their consent at any time.

A panel of user representatives have been consulted before and during the development of this study protocol. They have assessed and contributed to the study protocol and all written information given to the participants.

Sponsor will take out a drug liability insurance (LAF) to cover all participants.

Participants will not be charged for assessments or treatments under the study protocol. No financial compensation will be offered.

Source data including medical journals will be made available for monitoring and in the event of an inspection/audit from NOMA.

Financial support

Daratumumab is under patent for Janssen pharmaceutical company until 2025/26. The drug costs are considerable, but with the backing of ME/CFS support groups, we have funding for this pilot study for six patients, including lab and biobank investigations.

The ME/CFS research group at Haukeland University Hospital receives funding from the Kavli Trust. This pilot study will be performed with financial support from the patient organizations the Norwegian ME association, MENiN, MEandYou and Fokus på ME. The funding organizations have had no role in the study design.

Følsomhet Intern (gul)

Publication

The pilot study will be published in a peer-reviewed journal, regardless of the outcome of the trial.

References

1. Carruthers BM, Jain AK, De Meirleir KL, Peterson DL, Klimas NG, Lerner AM, et al. Myalgic encephalomyelitis/ chronic fatigue syndrome: clinical working case definition, diagnostic and treatment protocols. *J Chronic Fatigue Syndr.* 2003;11(1):7-36.
2. Nacul LC, Lacerda EM, Pheby D, Campion P, Molokhia M, Fayyaz S, et al. Prevalence of myalgic encephalomyelitis/chronic fatigue syndrome (ME/CFS) in three regions of England: a repeated cross-sectional study in primary care. *BMC Med.* 2011;9(1):91.
3. Valdez AR, Hancock EE, Adebayo S, Kiernicki DJ, Proskauer D, Attewell JR, et al. Estimating Prevalence, Demographics, and Costs of ME/CFS Using Large Scale Medical Claims Data and Machine Learning. *Front Pediatr.* 2018;6:412.
4. Falk Hvidberg M, Brinth LS, Olesen AV, Petersen KD, and Ehlers L. The Health-Related Quality of Life for Patients with Myalgic Encephalomyelitis / Chronic Fatigue Syndrome (ME/CFS). *PloS one.* 2015;10(7):e0132421.
5. Nacul LC, Lacerda EM, Campion P, Pheby D, Drachler Mde L, Leite JC, et al. The functional status and well being of people with myalgic encephalomyelitis/chronic fatigue syndrome and their carers. *BMC public health.* 2011;11(402).
6. Medicine Io. *Beyond Myalgic Encephalomyelitis/Chronic Fatigue Syndrome: Redefining an Illness*. Washington (DC): National Academies Press (US); 2015.
7. Albright F, Light K, Light A, Bateman L, and Cannon-Albright LA. Evidence for a heritable predisposition to Chronic Fatigue Syndrome. *BMC Neurol.* 2011;11:62.
8. Fluge O, and Mella O. Clinical impact of B-cell depletion with the anti-CD20 antibody rituximab in chronic fatigue syndrome: a preliminary case series. *BMC Neurol.* 2009;9(1):28.
9. Fluge O, Bruland O, Risa K, Storstein A, Kristoffersen EK, Sapkota D, et al. Benefit from B-Lymphocyte Depletion Using the Anti-CD20 Antibody Rituximab in Chronic Fatigue Syndrome. A Double-Blind and Placebo-Controlled Study. *PloS one.* 2011;6(10):e26358.
10. Fluge O, Risa K, Lunde S, Alme K, Rekeland IG, Sapkota D, et al. B-Lymphocyte Depletion in Myalgic Encephalopathy/ Chronic Fatigue Syndrome. An Open-Label Phase II Study with Rituximab Maintenance Treatment. *PloS one.* 2015;10(7):e0129898.
11. Fluge O, Rekeland IG, Lien K, Thurmer H, Borchgrevink PC, Schafer C, et al. B-Lymphocyte Depletion in Patients With Myalgic Encephalomyelitis/Chronic Fatigue Syndrome: A Randomized, Double-Blind, Placebo-Controlled Trial. *Ann Intern Med.* 2019;170(9):585-93.
12. Rekeland IG, Fosså A, Lande A, Ktoridou-Valen I, Sørland K, Holsen M, et al. Intravenous Cyclophosphamide in Myalgic Encephalomyelitis/Chronic Fatigue Syndrome. An Open-Label Phase II Study. *Front Med.* 2020;7:162.

Følsomhet Intern (gul)

13. Fluge Ø, Tronstad KJ, and Mella O. Pathomechanisms and possible interventions in myalgic encephalomyelitis/chronic fatigue syndrome (ME/CFS). *J Clin Invest.* 2021;131(14).
14. Lande A, Fluge Ø, Strand EB, Flåm ST, Sosa DD, Mella O, et al. Human Leukocyte Antigen alleles associated with Myalgic Encephalomyelitis/Chronic Fatigue Syndrome (ME/CFS). *Sci Rep.* 2020;10(1):5267.
15. Chang CM, Warren JL, and Engels EA. Chronic fatigue syndrome and subsequent risk of cancer among elderly US adults. *Cancer.* 2012;118(23):5929-36.
16. Lunde S, Kristoffersen EK, Sapkota D, Risa K, Dahl O, Bruland O, et al. Serum BAFF and APRIL Levels, T-Lymphocyte Subsets, and Immunoglobulins after B-Cell Depletion Using the Monoclonal Anti-CD20 Antibody Rituximab in Myalgic Encephalopathy/Chronic Fatigue Syndrome. *PloS one.* 2016;11(8):e0161226.
17. Sato W, Ono H, Matsutani T, Nakamura M, Shin I, Amano K, et al. Skewing of the B cell receptor repertoire in myalgic encephalomyelitis/chronic fatigue syndrome. *Brain, behavior, and immunity.* 2021.
18. Milivojevic M, Che X, Bateman L, Cheng A, Garcia BA, Hornig M, et al. Plasma proteomic profiling suggests an association between antigen driven clonal B cell expansion and ME/CFS. *PloS one.* 2020;15(7):e0236148.
19. Nguyen CB, Alsøe L, Lindvall JM, Sulheim D, Fagermoen E, Winger A, et al. Whole blood gene expression in adolescent chronic fatigue syndrome: an exploratory cross-sectional study suggesting altered B cell differentiation and survival. *J Transl Med.* 2017;15(1):102.
20. Scheibenbogen C, Loebel M, Freitag H, Krueger A, Bauer S, Antelmann M, et al. Immunoadsorption to remove beta2 adrenergic receptor antibodies in Chronic Fatigue Syndrome CFS/ME. *PloS one.* 2018;13(3):e0193672.
21. Hoel F, Hoel A, Pettersen IK, Rekeland IG, Risa K, Alme K, et al. A map of metabolic phenotypes in patients with myalgic encephalomyelitis/chronic fatigue syndrome. *JCI insight.* 2021;6(16).
22. Newton DJ, Kennedy G, Chan KK, Lang CC, Belch JJ, and Khan F. Large and small artery endothelial dysfunction in chronic fatigue syndrome. *International journal of cardiology.* 2012;154(3):335-6.
23. Sørland K, Sandvik MK, Rekeland IG, Ribu L, Småstuen MC, Mella O, et al. Reduced Endothelial Function in Myalgic Encephalomyelitis/Chronic Fatigue Syndrome-Results From Open-Label Cyclophosphamide Intervention Study. *Front Med (Lausanne).* 2021;8:642710.
24. Scherbakov N, Szklarski M, Hartwig J, Sotzny F, Lorenz S, Meyer A, et al. Peripheral endothelial dysfunction in myalgic encephalomyelitis/chronic fatigue syndrome. *ESC Heart Fail.* 2020.
25. Joseph P, Arevalo C, Oliveira RKF, Faria-Urbina M, Felsenstein D, Oaklander AL, et al. Insights From Invasive Cardiopulmonary Exercise Testing of Patients With Myalgic Encephalomyelitis/Chronic Fatigue Syndrome. *Chest.* 2021.
26. Vermeulen RC, and Vermeulen van Eck IW. Decreased oxygen extraction during cardiopulmonary exercise test in patients with chronic fatigue syndrome. *J Transl Med.* 2014;12:20.

Følsomhet Intern (gul)

27. Freeman R, and Komaroff AL. Does the chronic fatigue syndrome involve the autonomic nervous system? *Am J Med.* 1997;102(4):357-64.
28. Słomko J, Estévez-López F, Kujawski S, Zawadka-Kunikowska M, Tafil-Klawe M, Klawe JJ, et al. Autonomic Phenotypes in Chronic Fatigue Syndrome (CFS) Are Associated with Illness Severity: A Cluster Analysis. *J Clin Med.* 2020;9(8).
29. Germain A, Ruppert D, Levine SM, and Hanson MR. Metabolic profiling of a myalgic encephalomyelitis/chronic fatigue syndrome discovery cohort reveals disturbances in fatty acid and lipid metabolism. *Molecular bioSystems.* 2017;13(2):371-9.
30. Fluge O, Mella O, Bruland O, Risa K, Dyrstad SE, Alme K, et al. Metabolic profiling indicates impaired pyruvate dehydrogenase function in myalgic encephalopathy/chronic fatigue syndrome. *JCI insight.* 2016;1(21):e89376.
31. Rivera-Correa J, and Rodriguez A. Divergent Roles of Antiself Antibodies during Infection. *Trends Immunol.* 2018;39(7):515-22.
32. Skiba MA, and Kruse AC. Autoantibodies as Endogenous Modulators of GPCR Signaling. *Trends Pharmacol Sci.* 2021;42(3):135-50.
33. Li H, Kem DC, Reim S, Khan M, Vanderlinde-Wood M, Zillner C, et al. Agonistic autoantibodies as vasodilators in orthostatic hypotension: a new mechanism. *Hypertension.* 2012;59(2):402-8.
34. Fedorowski A, Li H, Yu X, Koelsch KA, Harris VM, Liles C, et al. Antiadrenergic autoimmunity in postural tachycardia syndrome. *Europace.* 2017;19(7):1211-9.
35. Loebel M, Grabowski P, Heidecke H, Bauer S, Hanitsch LG, Wittke K, et al. Antibodies to beta adrenergic and muscarinic cholinergic receptors in patients with Chronic Fatigue Syndrome. *Brain, behavior, and immunity.* 2016;52:32-9.
36. Cabral-Marques O, Marques A, Giil LM, De Vito R, Rademacher J, Günther J, et al. GPCR-specific autoantibody signatures are associated with physiological and pathological immune homeostasis. *Nat Commun.* 2018;9(1):5224.
37. Murphy WJ, and Longo DL. A Possible Role for Anti-idiotype Antibodies in SARS-CoV-2 Infection and Vaccination. *N Engl J Med.* 2021.
38. Chang HD, Tokoyoda K, Hoyer B, Alexander T, Khodadadi L, Mei H, et al. Pathogenic memory plasma cells in autoimmunity. *Curr Opin Immunol.* 2019;61:86-91.
39. Barnas JL, Looney RJ, and Anolik JH. B cell targeted therapies in autoimmune disease. *Curr Opin Immunol.* 2019;61:92-9.
40. Hogan KA, Chini CCS, and Chini EN. The Multi-faceted Ecto-enzyme CD38: Roles in Immunomodulation, Cancer, Aging, and Metabolic Diseases. *Front Immunol.* 2019;10:1187.
41. Piedra-Quintero ZL, Wilson Z, Nava P, and Guerau-de-Arellano M. CD38: An Immunomodulatory Molecule in Inflammation and Autoimmunity. *Front Immunol.* 2020;11:597959.
42. van de Donk NW, Janmaat ML, Mutis T, Lammerts van Bueren JJ, Ahmadi T, Sasser AK, et al. Monoclonal antibodies targeting CD38 in hematological malignancies and beyond. *Immunol Rev.* 2016;270(1):95-112.
43. Benfaremo D, and Gabrielli A. Is There a Future for Anti-CD38 Antibody Therapy in Systemic Autoimmune Diseases? *Cells.* 2019;9(1).

Følsomhet Intern (gul)

44. Rieger MJ, Stolz SM, Ludwig S, Benoit TM, Bissig M, Widmer CC, et al. Daratumumab in rituximab-refractory autoimmune haemolytic anaemia. *Br J Haematol.* 2021;194(5):931-4.
45. Pleguezuelo DE, Díaz-Simón R, Cabrera-Marante O, Lalueza A, Paz-Artal E, Lumbreras C, et al. Case Report: Resetting the Humoral Immune Response by Targeting Plasma Cells With Daratumumab in Anti-Phospholipid Syndrome. *Front Immunol.* 2021;12:667515.
46. Zaninoni A, Giannotta JA, Gallì A, Artuso R, Bianchi P, Malcovati L, et al. The Immunomodulatory Effect and Clinical Efficacy of Daratumumab in a Patient With Cold Agglutinin Disease. *Front Immunol.* 2021;12:649441.
47. Ostendorf L, Burns M, Durek P, Heinz GA, Heinrich F, Garantziotis P, et al. Targeting CD38 with Daratumumab in Refractory Systemic Lupus Erythematosus. *N Engl J Med.* 2020;383(12):1149-55.
48. Ehrenfeld M, Tincani A, Andreoli L, Cattalini M, Greenbaum A, Kanduc D, et al. Covid-19 and autoimmunity. *Autoimmun Rev.* 2020;19(8):102597.
49. Dotan A, and Shoenfeld Y. Post-COVID syndrome: the aftershock of SARS-CoV-2. *Int J Infect Dis.* 2021;114:233-5.
50. Chang SE, Feng A, Meng W, Apostolidis SA, Mack E, Artandi M, et al. New-onset IgG autoantibodies in hospitalized patients with COVID-19. *Nat Commun.* 2021;12(1):5417.
51. Wang EY, Mao T, Klein J, Dai Y, Huck JD, Jaycox JR, et al. Diverse functional autoantibodies in patients with COVID-19. *Nature.* 2021;595(7866):283-8.

Følsomhet Intern (gul)

Appendix 1: Safety

Safety monitoring

Safety assessment will consist of monitoring and recording all AEs by the principal investigator, including Serious Adverse Events (SAE), besides regular monitoring of hematological status. Any clinically significant change in a laboratory parameter will be defined as AE.

Adverse event/ serious adverse event

Definition of adverse events (AE)

An Adverse Event (AE) is any untoward medical occurrence in a patient administered a pharmaceutical product and which does not necessarily have to have a causal relationship with this treatment. It can therefore be any unfavorable and unintended sign, symptom, or disease temporally associated with the use of a medicinal product, whether or not considered related to the medicinal product. This includes any occurrence that is new in onset or aggravated in severity from the baseline condition, or abnormal results of any diagnostic procedures, including laboratory testing abnormalities.
All toxicity will be graded according to Common Terminology Criteria for Adverse Events (CTCAE) guidelines (version 5.0).
Adverse events will be graded using the following criteria:

- Grade 1 (Mild): discomfort noticed but no disruption of normal daily activity.
- Grade 2 (Moderate): discomfort sufficient to reduce or affect normal daily activity.
- Grade 3 (Severe): inability to work or perform normal daily activity.
- Grade 4 (Life-threatening or disabling): represents an immediate threat to life.

Furthermore, adverse events will be classified into serious adverse event (SAE) yes/no.

Definition and reporting of serious adverse events (SAE)

SAE is any clinical AE or abnormal laboratory test value that is serious and occurring during the course of the study, irrespective of the treatment received by the patient.
A SAE is any experience that suggests a significant hazard, contraindication, side effect or precaution. This includes any experience which:

- is fatal (note that death is an outcome, not an event).
- is life-threatening.
- requires in-patient hospitalization or prolongation of existing hospitalization.
- results in persistent or significant disability/incapacity.
- is a congenital anomaly/ birth defect.
- is medically significant or requires intervention to prevent one or more outcomes

Følsomhet Intern (gul)

listed below.

For reports of hospitalization, it is the sign, symptom, or diagnosis which led to hospitalization that is the serious event for which details must be provided. Any event requiring hospitalization or prolongation of hospitalization that occurs during the study must be reported as a SAE, except hospitalization of the following:

- hospitalization not intended to treat an acute illness or adverse event (e.g., social reasons, administration of study drug or study procedures).
- surgery or procedure planned before entry into the study (Note: hospitalization that were planned before the signing of consent form and where the underlying condition for which the hospitalization was planned has not worsened, will not be considered SAE. Any AE that results in a prolongation of the originally planned hospitalization is to be reported as a new SAE.

SAE must be reported to the sponsor within 24 hours of investigator/study nurse detecting the event or learning about it. Reporting is done through eCRF and by direct contact (by mail or telephone) to sponsor.

Suspected Unexpected Serious Adverse Reactions (SUSAR)

All **suspected** Adverse Reactions which occur in the trial and that are both **unexpected** and **serious**. Suspected adverse reactions (AR) are those AEs of which a reasonable causal relationship to any dose administered of the investigational medicinal product and the event is suspected. Unexpected adverse reactions are adverse reactions, of which the nature, or severity, is not consistent with the applicable product information (e.g.
Investigator's Brochure for an unapproved IMP or Summary of Product Characteristics (SPC) for an authorized medicinal product).
Such an event must be reported to the sponsor within 24 hours of investigator or study nurse detecting the event or learning about it. Reporting is done through the eCRF and by direct contact (by mail or telephone) to sponsor. SUSARS will be collected and reported to the competent authorities and ethics committees. If an SAE is deemed a SUSAR, reporting to NOMA will take place within 7 days (fatal or life-threatening SUSAR) or 15 days (other SUSARs) from receipt of notification of the event. A final report of SAEs will be submitted to the Norwegian Medicines Agency (NOMA) at the end of study.

Documenting assessment and reporting of adverse events

The investigator is responsible for ensuring that all AEs (including SAEs) that are observed or reported during the study, as outlined in the prior sections, are recorded on the eCRF. Adverse

Følsomhet Intern (gul)

events will be scored according to the NCI Common Terminology Criteria for Adverse Events, version 5.0 (See appendix VI). Investigator will assess the occurrence of AEs and SAEs at all subject evaluation time points during the study. All AEs and SAEs, whether volunteered by the subject, discovered by study staff during questioning, or detected through physical examination, laboratory testing, or other means, will be recorded in the subject`s medical record and on the AE eCRF.

Each recorded AE or SAE will be described by its duration, severity, suspected relationship to the investigational product, and any action taken.

Adverse events will be reported from the time of signature of informed consent until the end of study for the patient.

If an AE or SAE is present at the withdrawal visit or at the subject`s last participation in the study, it should be followed until the time of resolution or stabilized unless the subjects is lost to follow up. Resolution means the subject has returned to baseline state of health or the investigator does not expect any further improvement or worsening of the event.

Adverse events occurring after the end of follow-up in the study should also be reported if considered at least possibly related to the investigational medicinal product by the investigator.

Pre-existing conditions will be collected on the baseline concomitant diseases CRF, i.e., active (symptomatic) diseases of CTCAE grade 2, diseases under treatment, chronic diseases and long-term effects of past events as present at the time of baseline assessment.

All adverse events have to be reported with **the exception of**:

1. A pre-existing condition that does not increase in severity; the pre-existing condition should be reported on the baseline concomitant diseases CRF.
2. Any grade 1 adverse event.

Følsomhet Intern (gul)

Causality assessment of Serious Adverse Event

The investigator will decide whether the serious adverse events is related to the investigational treatment, i.e. daratumumab. The decision will be recorded on the serious adverse event report. The assessment of causality is made by the investigator using the following:

RELATIONSHIP	DESCRIPTION
UNRELATED	There is no evidence of any causal relationship
UNLIKELY	There is little evidence to suggest there is a causal relationship (e.g., the event did not occur within a reasonable time after administration of the trial medication).<br>There is another reasonable explanation for the event (e.g., the patient's clinical condition, other concomitant treatments).
POSSIBLE	There is some evidence to suggest a causal relationship (e.g., because the event occurs within a reasonable time after administration of the trial medication).<br>However, the influence of other factors may have contributed to the event (e.g., the patient's clinical condition, other concomitant treatments).
PROBABLE	There is evidence to suggest a causal relationship and the influence of other factors is unlikely.
DEFINITELY	There is clear evidence to suggest a causal relationship and other possible contributing factors can be ruled out.
NOT ASSESSABLE	There is insufficient or incomplete evidence to make a clinical judgment of the causal relationship.

Follow up of Serious Adverse Events

All serious adverse events will be followed clinically until they are resolved or until a stable situation has been reached. Depending on the event, follow up may require additional tests or medical procedures as indicated, and/or referral to the general physician or a medical specialist. Follow up information on SAEs should be reported regularly in the eCRF until recovery or until a stable situation has been reached. The final outcome of the SAE should be reported on a final SAE report.

Følsomhet Intern (gul)
