# Supplementary figures and images for: Plasma cell targeting with the anti-CD38 antibody daratumumab in myalgic encephalomyelitis/chronic fatigue syndrome—a clinical pilot study
Source: Front Med (Lausanne). 2025 Jul 9;12:1607353. doi: 10.3389/fmed.2025.1607353 (PMC12283730; doi:10.3389/fmed.2025.1607353)

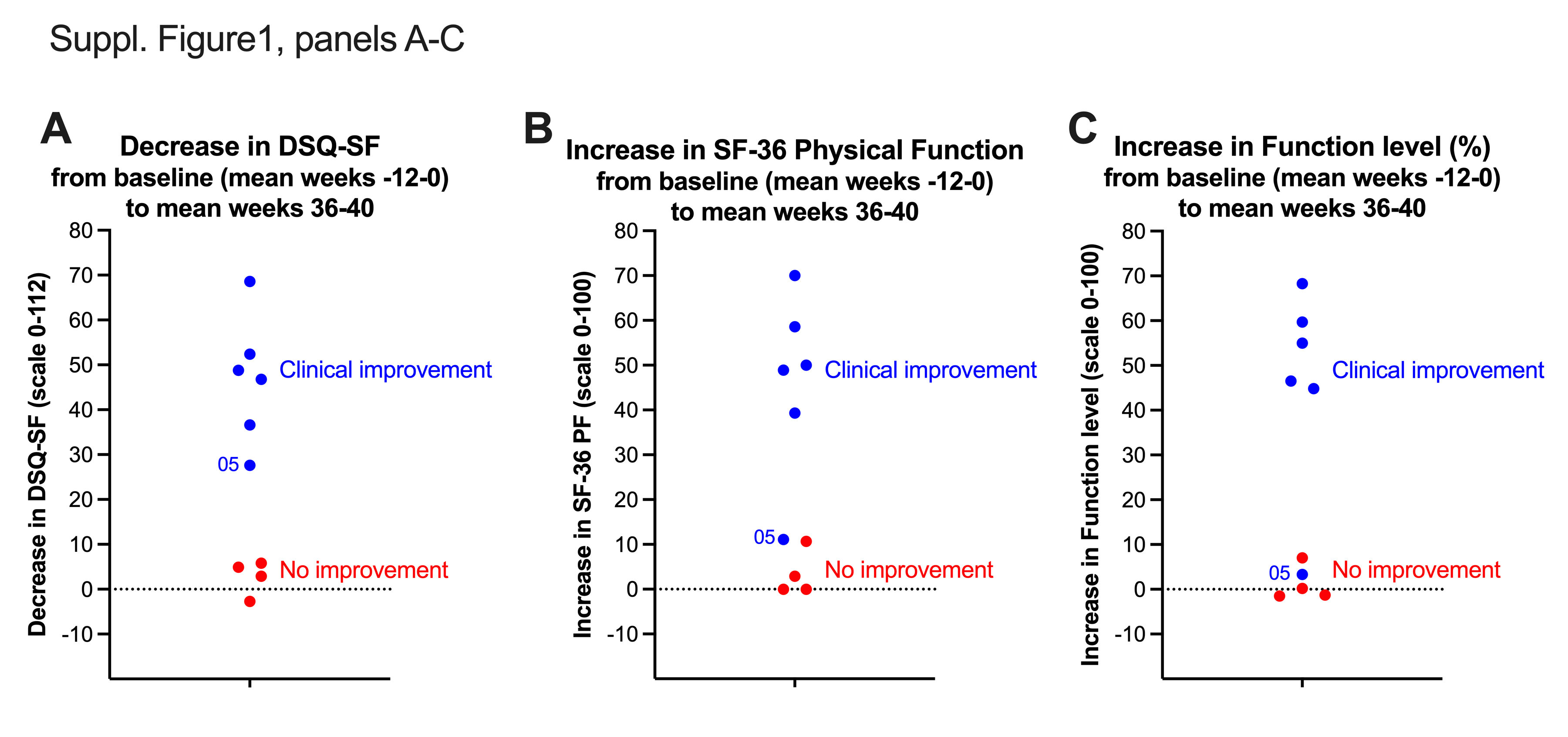

Supplement: Supplementary file 3 [file Image_1.TIFF]

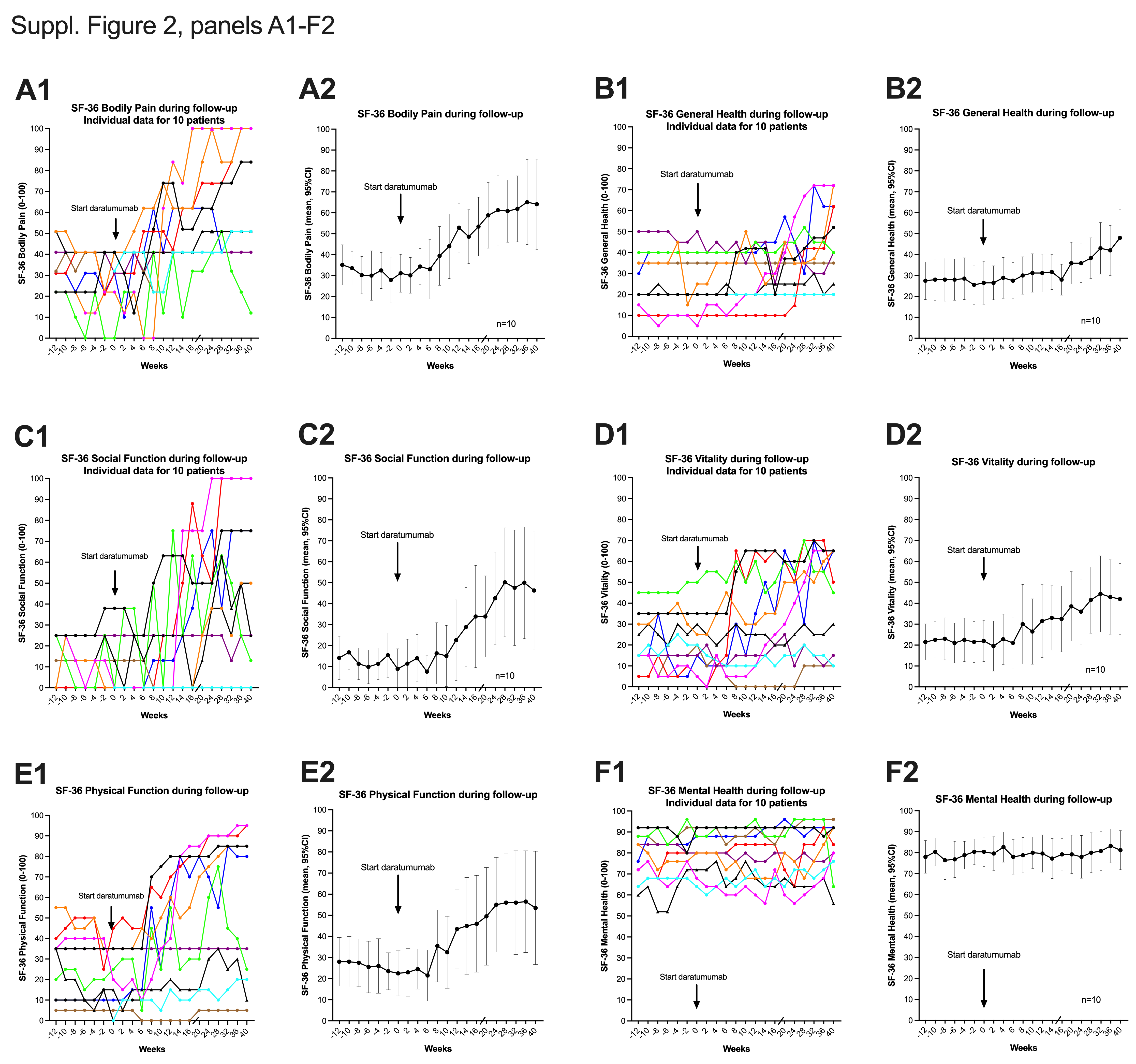

Supplement: Supplementary file 4 [file Image_2.TIFF]

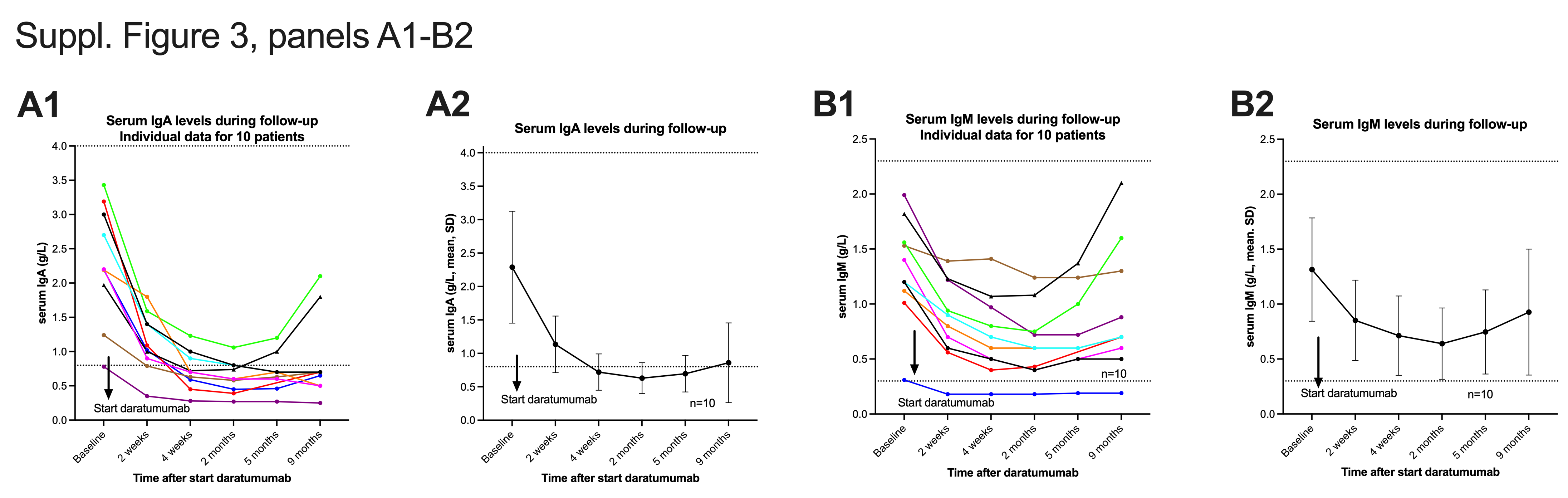

Supplement: Supplementary file 5 [file Image_3.TIFF]

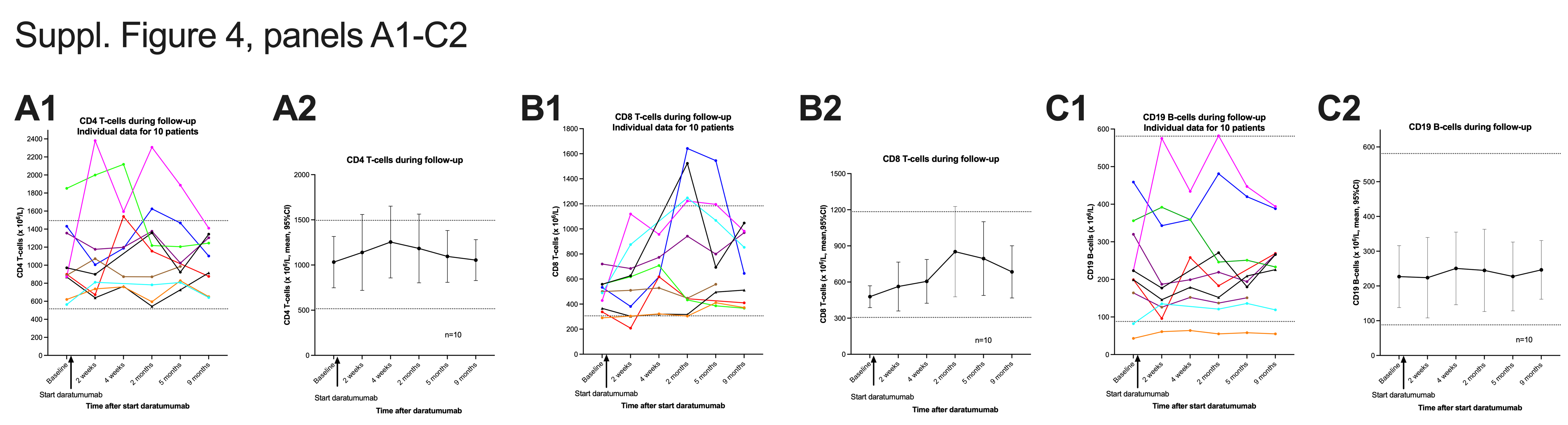

Supplement: Supplementary file 6 [file Image_4.TIFF]
